# Characterization of the Membrane-Associated Electron-Bifurcating Flavoenzyme EtfABCX from the Hyperthermophilic Bacterium *Thermotoga maritima*

**DOI:** 10.1021/acs.biochem.3c00473

**Published:** 2023-12-07

**Authors:** Xiaoxuan Ge, Gerrit J. Schut, Jessica Tran, Farris L. Poole II, Dimitri Niks, Kevin Menjivar, Russ Hille, Michael W. W. Adams

**Affiliations:** † Department of Biochemistry and Molecular Biology, University of Georgia, Athens, Georgia 30602, United States; ‡ Department of Biochemistry, University of California, Riverside, Riverside, California 92507, United States

## Abstract

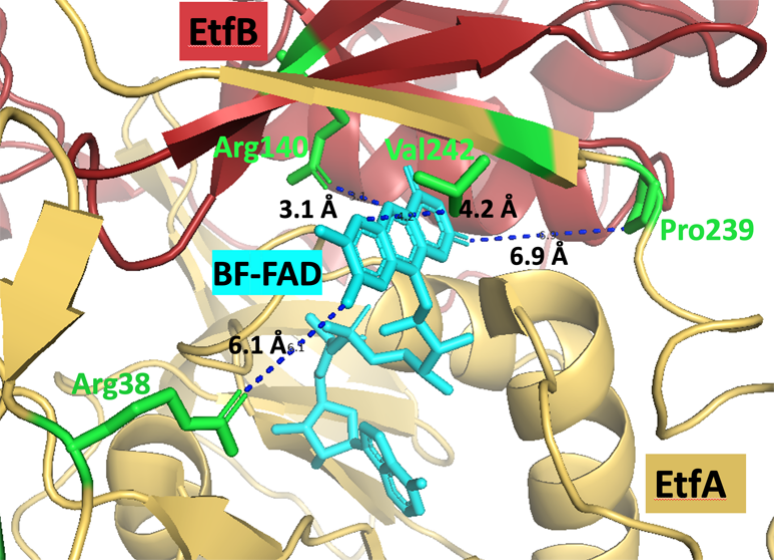

Electron bifurcation is an energy-conservation mechanism in which a single enzyme couples an exergonic reaction with an endergonic one. Heterotetrameric EtfABCX drives the reduction of low-potential ferredoxin (*E*°′ ∼ −450 mV) by oxidation of the midpotential NADH (*E*°′ = −320 mV) by simultaneously coupling the reaction to reduction of the high-potential menaquinone (*E*°′ = −74 mV). Electron bifurcation occurs at the NADH-oxidizing bifurcating-flavin adenine dinucleotide (BF-FAD) in EtfA, which has extremely crossed half-potentials and passes the first, high-potential electron to an electron-transferring FAD and via two iron–sulfur clusters eventually to menaquinone. The low-potential electron on the BF-FAD semiquinone simultaneously reduces ferredoxin. We have expressed the genes encoding*Thermotoga maritima*EtfABCX in *E. coli* and purified the EtfABCX holoenzyme and the EtfAB subcomplex. The bifurcation activity of EtfABCX was demonstrated by using electron paramagnetic resonance (EPR) to follow accumulation of reduced ferredoxin. To elucidate structural factors that impart the bifurcating ability, EPR and NADH titrations monitored by visible spectroscopy and dye-linked enzyme assays have been employed to characterize four conserved residues, R38, P239, and V242 in EtfA and R140 in EtfB, in the immediate vicinity of the BF-FAD. The R38, P239, and V242 variants showed diminished but still significant bifurcation activity. Despite still being partially reduced by NADH, the R140 variant had no bifurcation activity, and electron transfer to its two [4Fe-4S] clusters was prevented. The role of R140 is discussed in terms of the bifurcation mechanism in EtfABCX and in the other three families of bifurcating enzymes.

## Introduction

Electron bifurcation is an energy-coupling mechanism that combines exergonic and endergonic electron-transfer events to efficiently utilize enzymatic energy to maximize cellular yields.^1,2^ Electron-bifurcating enzymes are found in most anaerobic microorganisms where they are used to generate the low-potential reducing equivalents (e.g., reduced ferredoxin) required in fundamental pathways such as H_2_, methane, and butyrate metabolism as well as fixation of both nitrogen and carbon;^1,3−5^ they are also part of some aerobic respiratory chains.^6^ An electron bifurcation mechanism is essential for autotrophic microbes and important for heterotrophs grown on certain substrates.^2,5^ Electron bifurcation is referred to as a primary energy-conservation mechanism along with oxidative phosphorylation and substrate-level phosphorylation.^7,8^

Bifurcating enzymes contain a flavin adenine dinucleotide (FAD) or flavin mononucleotide (FMN) that splits electron pairs from a midpotential donor and transfers them to separate high- and low-potential acceptors in a tightly coupled manner. A key element of all bifurcating flavins is that they possess highly crossed half-potentials, with the semiquinone/hydroquinone couple being much higher than the quinone/semiquinone couple. The first, high-potential electron out of the fully reduced bifurcating flavin once it has been reduced is sent along a high-potential pathway to reduce the high-potential acceptor, while the remaining low-potential and highly thermodynamically unstable electron on the bifurcating flavin is used to reduce, e.g., ferredoxin.^1,3,5^ Thus, with each bifurcating event, one electron is transferred to the high-potential pathway and another to ferredoxin. Moreover, the enzyme need not become fully reduced before ferredoxin reduction occurs. Four types of phylogenetically unrelated bifurcating systems have been identified. These include the electron-transferring flavoprotein oxidoreductase (Etf) family sometimes named Fix for its role in nitrogen-fixing organisms (e.g., Fix/EtfABCX),^9^ NADH-dependent reduced ferredoxin/NADP^+^ oxidoreductase (NfnAB),^10^ hydrogenase/heterodisulfide reductase (Hdr-Mvh),^11^ and the Bfu family of bifurcating enzymes that have a conserved bifurcating core where FeFe and NiFe hydrogenases are the prototypes.^5^ The EtfABCX complex uses NADH (*E*_m_ = −320 mV) to reduce ferredoxin (*E*_m_ ∼ −450 mV) by coupling the reaction to the exergonic reduction of menaquinone (MQ, *E*_m_ = −74 mV; eq 1),^5,6,9^ while the NfnAB complex catalyzes the reduction of NADP^+^ for biosynthesis (E’ ∼ −380 mV under physiological conditions) by NADH by coupling the oxidation of reduced ferredoxin.^10,12^ In a similar fashion, the Hdr-Mvh complex contains hydrogenase (MvhAGD) and heterodisulfide reductase (HdrABC) and uses H_2_ (*E*°′ = −414 mV) to reduce a heterodisulfide (*E*_m_ = −140 mV) and ferredoxin,^13^ while the FeFe and NiFe-Bfu hydrogenases couple endergonic NADH oxidation and exergonic oxidation of ferredoxin to reduce protons and produce H_2_.^5,14^

A detailed oxidation–reduction and spectroscopic analysis of the BF-FAD in NfnAB has revealed that the redox potentials of its quinone (Q)/semiquinone (SQ) and SQ/hydroquinone (HQ) couples are crossed such that the potential of the Q/SQ couple is highly reducing (*E*_m_ = −911 mV) and more than sufficient to drive reduction of ferredoxin.^15^ A similar mechanism is thought to occur in BF-FAD found in EtfAB and HdrABC electron-bifurcating complexes. The situation is very different in members of the Bfu family, as these lack this conventional BF-FAD and instead contain a highly conserved arrangement of FMN and three iron–sulfur clusters. The mechanism of electron bifurcation in the Bfu family is still not fully understood.^1,3,5^
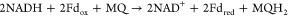
1

FixABCX was first characterized from the mesophilic N_2_-fixing bacterium *Azotobacter vinelandii* and shown to couple NADH oxidation (*E*_m_ = −320 mV) to the simultaneous reduction of coenzyme Q (*E*_m_ = 10 mV) and flavodoxin (*E*_m_ = −460 mV), providing the cell with low-potential electrons to reduce N_2_ to ammonia.^6^ The homologous EtfABCX from the non-N_2_-fixing hyperthermophilic archaeon *Pyrobaculum aerophilum* was subsequently obtained by heterologously expressing the holoenzyme in the hyperthermophilic archaeon *Pyrococcus furiosus*.^8^ Using results from a combination of transient absorption, EPR, and ultraviolet visible (UV/vis) titrations of *P. aerophilum* EtfABCX, a catalytic cycle was proposed involving the formation of an intermediate NAD^+^-bound complex.^8^ Importantly, even though EtfABCX and NfnAB have a similar bifurcating site, namely, the BF-FAD, their catalytic cycles are very different, specifically in the role of NAD^+^, the nature of the resting and bifurcating-ready states of the enzymes, how electron flow is gated, and in the two two-electron cycles constituting the overall four-electron reaction.^8^

**Figure 1:**
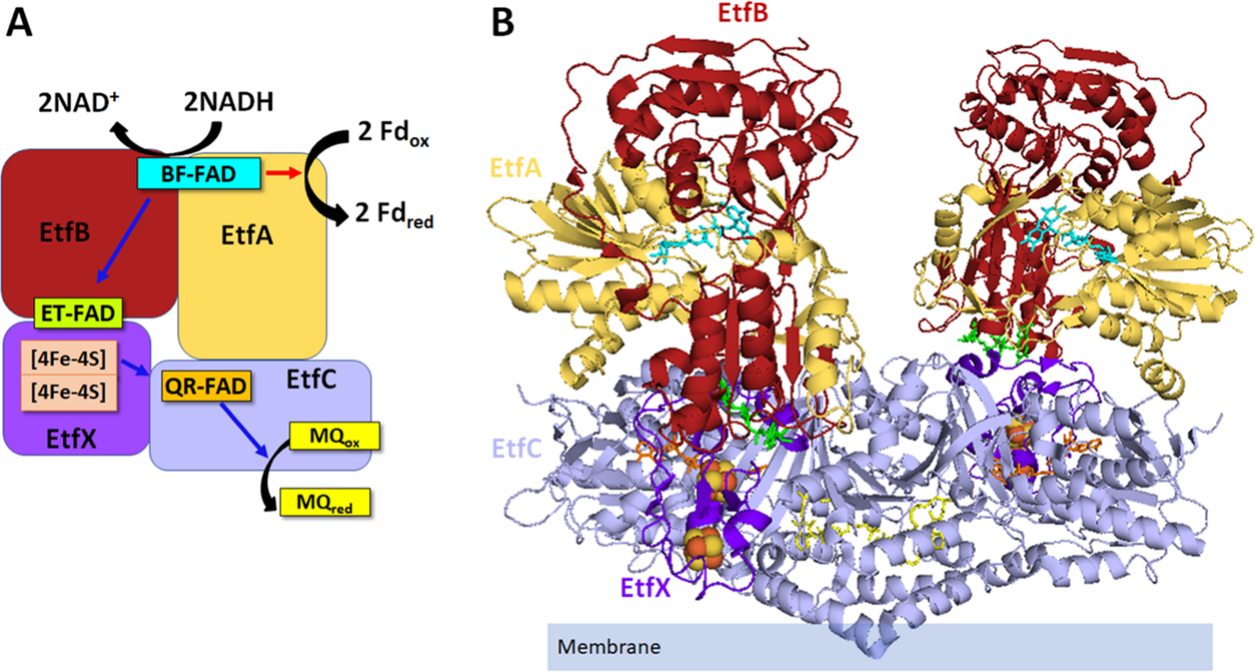
Model and ribbon diagram of *T. maritima* EtfABCX Cryo-EM structure (PDB: 7KOE). (A) Model of EtfABCX. Red arrow indicates the endergonic branch, and blue arrows indicate the exergonic branch. (B) Structure of EtfABCX super-tetramer (PDB: 7KOE). EtfA, EtfB, EtfC, and EtfX were in yellow, red, light purple, and purple, respectively. BF-FAD, ET-FAD, QR-FAD, and menaquinone were highlighted in cyan, green, orange, and yellow in panels (A, B). Two [4Fe-4S] clusters were colored pink in panel (A) and represented as balls in panel (B) in EtfX subunits.

*P. aerophilum* EtfABCX and the bacterial enzyme EtfAB-bcd (butyryl-CoA dehydrogenase) have a common EtfAB subcomplex. In EtfABCX, EtfA contains the BF-FAD and EtfB contains a conventional electron-transferring or ET-FAD.^2^ The ET-FAD can be removed from both subcomplexes and the potentials of the Q/SQ and SQ/HQ of the resulting BF-FAD become uncrossed, which is thought to be due to a specific conformational change.^16^ The cryo-EM structure of EtfABCX from the hyperthermophilic anaerobic bacterium *T. maritima*,^9^ which also does not fix N_2_, showed that the structure of the EtfAB subcomplex of EtfABCX was, as expected, highly similar to that found in EtfAB-bcd and related enzymes.^17,18^ In addition to the BF-FAD in EtfA and ET-FAD in EtfB, the *T. maritima* EtfABCX complex contains two [4Fe-4S] clusters in EtfX and a third FAD in EtfC.^6,8^ From the structure, EtfAB was predicted to shuttle high-potential electrons from the BF-FAD to reduce the quinone via the two [4Fe-4S] clusters in EtfX and the so-called quinone-reducing or QR-FAD in EtfC. The structure indicated that low-potential electrons are likely transferred directly to ferredoxin from the BF-FAD (Figure 1). The structure also revealed that *T. maritima* EtfABCX is a dimer of EtfABCX protomers, (EtfABCX)_2_, with extensive intermolecular interactions via two membrane-associated EtfC subunits that also contain bound menaquinone (Figure 1). This arrangement of redox-active cofactors necessitated a revision in the catalytic mechanism of EtfABCX^9^ in which the iron–sulfur clusters are part of the high-potential pathway leading to menaquinone reduction, with ferredoxin reduced directly by the transiently formed BF-FAD^•–^.

The cryo-EM structure of *T. maritima* EtfABCX was previously obtained using holoenzyme that was heterologously expressed in *P. furiosus*,^9^ but the very low yield precluded catalytic and spectroscopic analyses. The goals of the present study were therefore to optimize the expression of the *T. maritima* EtfABCX enzyme in *E. coli* and obtain high yields of both the holoenzyme and the EtfAB subcomplex in order to investigate the ferredoxin- and NADH-dependent bifurcation activity using EPR and UV/vis spectroscopy. In particular, using EPR spectroscopy allows us to monitor for the first time the redox states of the three iron–sulfur clusters involved in the overall bifurcation reaction (two in EtfX and one in ferredoxin). In addition, the roles of key amino acid residues in the vicinity of the BF-FAD in imparting the bifurcating activity would then be investigated through site-directed mutagenesis. Our results show that Arg140 in *T. maritima* EtfB plays a crucial role in electron bifurcation by BF-FAD and that a potential mechanism is proposed. Meanwhile, the availability of recombinant *T. maritima* EtfABCX has also allowed a detailed analysis by anaerobic small-angle X-ray scattering (SAXS) of the conformational equilibria it assumes in solution and the effects of NAD(H) binding.^19^

## Materials/Experimental Details

### Expression and Purification of Recombinant *T. maritima* EtfABCX and EtfAB

The EtfABCX operon was amplified by polymerase chain reaction (PCR) from plasmid pGL093 that was used for its expression in *P. furiosus*.^9^ The PCR amplicons were cloned into the pET-21a(+) plasmid (Novagen) by Gibson assembly (New England Biolabs). The EtfABCX operon was under the control of the T7 promoter, and an N-terminal 9× His tag with flanking Ala spacers was encoded on the 5′ end of *etfA*. The resulting plasmid pET-21a(+)-*etfABCX* was sequence-verified and was used to transform the expression strain *E. coli* BL21 (DE3) Δ*iscR* with the plasmid pLysS (Novagen) for rare codon expression.^20^ The recombinant strain was grown anaerobically in LB media supplemented with ampicillin (50 μg/mL), chloramphenicol (30 μg/mL), ferrous ammonium sulfate (100 μM), cysteine (100 μM), glucose (0.5%, w/v), fumarate (0.5%, w/v), and riboflavin (0.1 mg/L) in a 20 L fermenter with N_2_/CO_2_ as the flushing gas. Protein expression was induced at an OD_600_ ∼ 0.6 with 0.5 mM IPTG and the temperature was reduced from 37 to 30 °C. The cells were harvested after 16 h and resuspended in an anaerobic starting buffer (phosphate 50 mM, pH 7.5, 500 mM NaCl, 5 mM imidazole). The cells were lysed by sonication and centrifuged to remove unlysed cells. The supernatant fractions were loaded onto a HisTrap FF crude column (GE Healthcare) pre-equilibrated with the starting buffer and washed with two column volume of wash buffer (50 mM phosphate, pH 7.5, containing 500 mM NaCl and 30 mM imidazole), and proteins were then eluted with an elution buffer (50 mM phosphate, pH 7.5, containing 500 mM NaCl and 300 mM imidazole). Fractions were collected and further purified by gel filtration using a Superdex 200 HiLoad 26/60 prep-grade column (GE Healthcare) equilibrated with HEPES 25 mM, pH 7.5, containing 200 mM NaCl. Fractions containing the purified EtfABCX or EtfAB were determined by a sodium dodecyl sulfate-polyacrylamide gel (SDS-PAGE). All purification steps starting from cell lysis were performed under anaerobic conditions and all buffers used in the purification were thoroughly degassed and sealed in airtight bottles under argon gas. All variants were purified following the same method described above. Anaerobic purification results in partial reduction of as-isolated wild-type and variant EtfABCX (as observed by EPR; data not shown). The level of reduction is preparation-dependent. All primer details are listed in Table S1. EtfAB protein concentrations were estimated by the Bradford assay, while EtfABCX protein concentrations were first estimated by Bradford assay and then corrected by amino acid analysis (AAA Service Laboratory, Inc., Boring, Oregon) performed on the WT EtfABCX. The subunits of the recombinant EtfAB and EtfABCX complexes were verified by tandem mass spectrometry (MS/MS) at the Proteomics and Mass Spectrometry Facility at the University of Georgia.

### Expression and Purification of Recombinant *T. maritima* Ferredoxin

The *T. maritima* ferredoxin was amplified by PCR from genomic DNA and cloned into the pET-24a(+) plasmid (Novagen) by Gibson assembly (Table S1). The resulting plasmid pET-24a(+)-*Fd* was sequence-verified and used to transform the expression strain *E. coli* BL21 (DE3) with the plasmid pLysS (Novagen).^20^ The recombinant strain was grown anaerobically in LB media supplemented with kanamycin (50 μg/mL), ferrous ammonium sulfate (500 μM), cysteine (500 μM), glucose (0.5%, w/v), and fumarate (0.5%, w/v) in a 20 L fermenter. Protein expression was induced at an OD_600_ ∼ 0.6 by addition of 0.5 mM IPTG and the temperature was reduced from 37 to 30 °C. Cells were harvested after 16 h and resuspended in the anaerobic buffer (HEPES 25 mM, pH 7.5). Ferredoxin was purified anaerobically from the cytoplasmic fraction using a custom 100 mL DEAE FF column and a Superdex S75 HiLoad 26/60 prep-grade column (GE Healthcare) and the homogeneity of the sample was confirmed by SDS-PAGE (Figure S1).

### Site-Specific Mutations of EtfABCX and EtfAB

All *etfABCX* variants were generated based on the wild-type plasmid pET-21a(+)-*etfABCX* using Site-directed, Ligase-Independent Mutagenesis (SLIM) developed by Chiu et al.^21^ The primers that were used are given in Table S1. All mutated plasmids were sequence-verified and transformed into *E. coli* BL21 (DE3)ΔiscR for protein overexpression and purification.

### Multialignment and Phylogenetic Analysis of *T. maritima* EtfA and EtfB

Homologue sequences of EtfA and EtfB were selected from bifurcating enzymes based on the five groups of phylogenetic analysis in the study of Costas et al.^16^ plus sequences that have published structures (PDB: 5OL2,^17^4KPU,^22^7QH2,^23,24^6FAH,^18^1EFV,^25^1EFP,^26^1O94,^27^5OW0,^28^3IH5, 3FET) or mutations of interest;^29^ details of these sequences can be found in Tables S2A and S2B. Sequences of 43 EtfA homologues (β subunits) and 42 EtfB homologues (α subunits) were first aligned individually using Clustal Omega (https://www.ebi.ac.uk/Tools/msa/clustalo/),^30^ then the two resultant alignment blocks were concatenated by Geneious Prime following the names of the β subunit sequences. A phylogenetic tree was made using Geneious Prime by Jukes-Cantor genetic distance model and the neighbor-joining method; a consensus tree was built here using Bootstrap as the resampling method, and the number of replicates was 100.

### Quantification of FAD and Iron

For FAD, each EtfABCX/AB complex was diluted to about 1 mg/mL using HEPES buffer (50 mM HEPES, pH 7.5, 100 mM NaCl) and denatured by 1% SDS at room temperature for 10 min to release FAD. Samples were transferred into glass cuvettes and their absorbance from 200 to 800 nm was scanned by a Cary 100 UV–vis spectrophotometer (Agilent) at room temperature. The extinction coefficient ε_450_ = 11.3 mM^–1^ cm^–1^ was used to calculate free FAD. The bathophenanthroline method^31^ was used to determine iron concentrations in EtfABCX. Specifically, 250 μL of 10 μM protein was denatured by 0.01× concentrated HCl at 80 °C, 10 min ddH_2_O (750 μL) was then added to dilute each sample, followed by 50 μL of 10% hydroxylamine-HCl and 250 μL of 0.1% (w/v) bathophenanthroline in the same order. After 1 h, the absorbance of each sample was measured spectrophotometrically at 535 nm. Concentrations of each sample were determined by a standard curve ranging from 0 to 500 μM.

### Enzyme Assays

Dye-linked nonbifurcating activities of EtfABCX/AB complexes were measured anaerobically at 75 °C in 3 mL glass cuvettes sealed with rubber stoppers containing 50 mM HEPES, pH 7.5, 100 mM NaCl, 0.5 mM NADH, 0.2 mM iodonitrotetrazolium chloride, and about 15 μg of EtfABCX or EtfAB protein. Reactions were initiated by the injection of NADH into the cuvettes. The formation of red formazan was followed at 500 nm in a Cary 100 UV–vis spectrophotometer with a Peltier-based temperature controller (Agilent). All data were normalized by the amount of protein that was used. In the investigation of effects of temperature, pH, and salt, the corresponding values were changed accordingly. Temperatures ranged from about 20 to 90 °C in the temperature effect assay. For pH effects, different buffers were used for proper buffering range: 50 mM acetate and 100 mM NaCl for pH 5; 50 mM phosphate buffer and 100 mM NaCl for pH 6, 6.5, 7, 7.5, and 8; and 50 mM CHES and 100 mM NaCl for pH 9 and 10.

### EPR Spectroscopy

All EPR samples were prepared in 50 mM HEPES, 200 mM NaCl, pH 7.5 buffer at 25 °C in 4 mm precision quartz (PQ) EPR tubes. All EPR samples were made with air-oxidized *T. maritima* EtfABCX. The enzyme was air-oxidized via thorough mixing with a pipet for ∼10 s. A quantification standard sample was made using 21.4 μM dithionite-reduced *T. maritima* ferredoxin monitored at 390 nm (using a Hewlett-Packard 8452A diode array spectrophotometer with a custom holder for the EPR tube) to ensure complete reduction. Experimental samples (ca. 220 μL), initially aerobic, were made anaerobic by addition of catalytic amounts of glucose oxidase from *Acidaminococcus niger* and bovine liver catalase along with 2–10 mM of glucose prior to injection into the Ar-flushed septa-sealed EPR tubes. The samples were then incubated for 30 min in the dark (to limit the amount of photoreduction) to allow for oxygen scavenging. NADH stock solutions were made anaerobic using the Ar train at room temperature and were then injected into their respective EPR sample tubes through the use of another anaerobic Hamilton syringe. Sample reduction via NADH was monitored at 426 nm to minimize the effect of the presence of large excess of NADH. Once it was determined that the reaction had come to completion, the EPR tubes containing the samples were frozen in an ethanol/dry ice bath. To ensure reproducibility, samples containing ferredoxin were duplicated. A reliable and accurate estimate of the amount of ferredoxin reduction was performed by, first, obtaining a difference spectrum by subtracting the spectrum of NADH-reduced EtfABCX from the spectrum of NADH-reduced EtfABCX in the presence of ferredoxin. The resultant difference spectrum was then compared to the 21.4 μM reduced ferredoxin standard. The ratio of intensities at the *g*_1_ of the difference spectrum and of the reduced ferredoxin standard was used to determine the amount of ferredoxin reduced. EPR experiments were performed with a Bruker Magnettech ESR 5000 fitted with a LTR-MS-5000 liquid helium cryostat (Advanced Research Systems) and a Lakeshore 335 variable temperature controller set at 10 K with a modulation amplitude of 1 mT and a power of 0.1 mW, unless otherwise stated. Acquisition software, ESRStudio 1.80.0, was used to baseline correct and process spectra as well as for the manipulation of spectra to obtain the amount of ferredoxin reduced throughout the course of the experiments. EPR simulations were performed using EasySpin version 5.2.35.^32^ The spectra shown represent an average of four separate scans. The *g*-strain for g_3_ of [4Fe–4S]-I was fixed during simulations of the wild-type EtfABCX. For simulations of the variants, the *g*-values obtained from the simulation of the wild-type spectrum were fixed, and only the relative contributions of the individual species to the composite spectrum were allowed to vary.

### Reductive Titration

Titrations were performed at 25 °C in an upcycled Agilent HP 8453 operating under OlisWorks (Olis) using 600 μL of masked quartz cuvettes (Starna) anaerobically sealed with silicon stoppers. The reductant was anaerobically added in small quantities with a 10 μL gastight syringe (Hamilton) to samples prepared in 50 mM HEPES, pH 7.5, with 200 mM NaCl. UV–vis spectra (200–1000 nm) were recorded after each addition. All solutions were prepared under strict anaerobic conditions, and stock solutions of titanium citrate were prepared as described previously.^8^

To focus on only one flavin undergoing reduction in each phase, difference spectra were used. In these, the final spectrum of each phase was subtracted from each other spectrum in the phase, and the three phases were identified by noting the different changes in A374 characterizing initial reduction of FAD to FAD^•–^, FAD^•–^ to FADH_2_, and FAD to FADH_2_.

## Results

### Heterologous Production and Characterization of EtfABCX and EtfAB from *T. maritima*

The *T. maritima* EtfABCX operon was cloned into the pET-21a (+) plasmid by Gibson assembly with an N-terminal 9× His tag on the 5′ end of *etfA* (Table S1). The *E. coli*BL21(DE3) pLysS Δ*iscR* strain was used for overexpression and was grown anaerobically in a 20 L fermenter in a medium containing excess Fe and cysteine (for Fe–S cluster biosynthesis) and riboflavin (for FAD biosynthesis). Even though *T. maritima* EtfABCX used menaquinone as a substrate and was predicted to be membrane-associated, it was located in the cytoplasmic fraction of *E. coli* cell extracts and was stable in solution without the addition of any detergent. Both EtfABCX and EtfAB complexes were purified by a two-step purification involving affinity chromatography and by a gel filtration column under anaerobic conditions. Analysis of purified recombinantly expressed EtfABCX by size exclusion chromatography revealed two major peaks, corresponding to the octomeric superdimer (EtfABCX)_2_ holoenzyme (256 kDa) and the heterodimeric EtfAB subcomplex (68 kDa) with a minor peak corresponding to the 16-subunit super-tetramer (EtfABCX)_4_ (512 kDa; Figure S1A). The EtfABCX holoenzyme and EtfAB subcomplex forms were obtained in comparable amounts, which is in contrast to when the enzyme was heterologously produced in *P. furiosus* where no significant amounts of the EtfAB subcomplex were detected.^9^ Meanwhile, *T. maritima* ferredoxin (6.2 kDa) was also overexpressed in *E. coli* BL21 (DE3) pLysS and purified using a two-step purification involving affinity chromatography and anaerobic gel filtration (Figure S1B).

*T. maritima* EftABCX contained 7.1 ± 0.5 Fe atoms and 3.26 ± 0.14 FADs by direct colorimetric analysis and visible absorption, respectively (see Tables S3 and S4). Using the same methods, EtfAB contained 1.79 ± 0.9 FADs, but iron was beyond detection due to the lack of the EtfX subunit. The recombinant holoenzyme produced in *E. coli* therefore has a complete cofactor content of two [4Fe-4S] clusters (in EtfX) and three FADs (BF-, ET-, and QR-FAD in EtfA, EtfB, and EtfC, respectively; Figure 1A). EtfABCX catalyzed a nonbifurcating dye-linked assay in which the oxidation of NADH is coupled to the reduction of the artificial electron acceptor iodonitrotetrazolium. The optimum temperature for this activity for EtfABCX was between 70 and 80 °C (at pH 7.5; Figure S2A), consistent with the optimum growth temperature of *T. maritima,*(33) and at 75 °C, the optimum pH was broad, ranging from 7.5 to 9.0 (Figure S2B).

**Figure 2:**
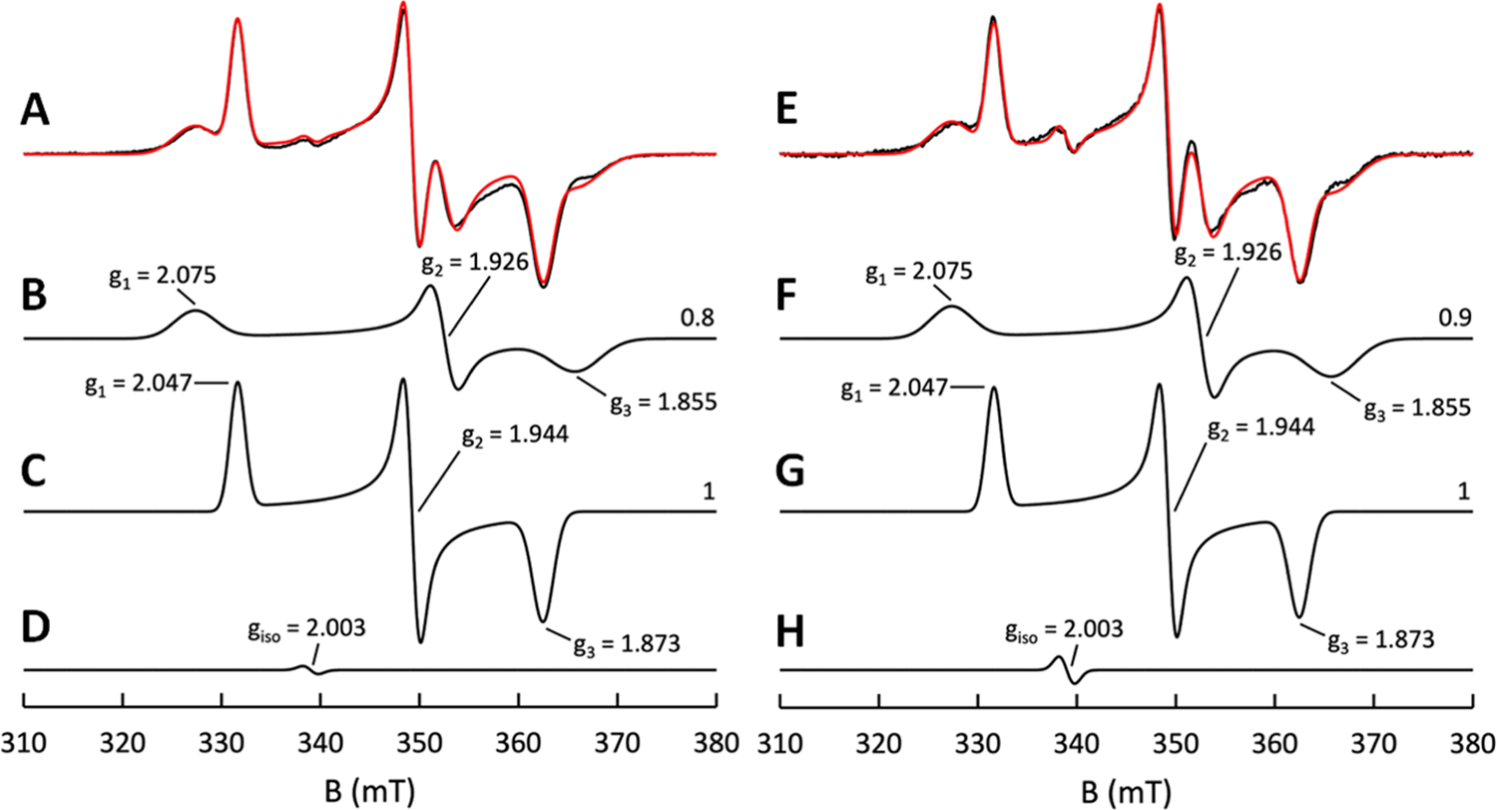
Deconvolution of the EPR signals seen from the dithionite-reduced EtfABCX wild-type and the R140Q mutant. EtfABCX wild-type: (A) experimental spectrum (black) and corresponding simulation (red) seen with dithionite-reduced EtfABCX. Samples contained 23.1 μM EtfABCX in which the enzyme was mixed with the substrate at room temperature and frozen in a dry ice/ethanol bath after determining full reduction via UV/vis spectroscopy (approximately 5 min). The individual components of the simulated spectra attributed to EtfABCX are (B) [4Fe-4S]-I cluster, (C) [4Fe-4S]-II cluster, and (D) anionic semiquinone. EtfABCX R140Q: (E) experimental spectrum (black) and corresponding simulation (red) seen with dithionite-reduced EtfABCX R140Q. The individual components of the simulated spectra attributed to EtfABCX are (F) [4Fe-4S]-I cluster, (G) [4Fe-4S]-II cluster, and (H) anionic semiquinone. The values on the right side of the individual component spectra correspond to the relative contributions of the said component to the overall spectra. Samples were made in 50 mM HEPES, 200 mM NaCl, pH 7.5 buffer.

Analysis by EPR spectroscopy of EtfABCX reduced by excess sodium dithionite revealed a complex spectrum at 10 K that could be assigned to two noninteracting reduced [4Fe-4S] clusters and an anionic semiquinone, FAD^•–^, which can be attributed to the one-electron-reduced ET-FAD (Figure 2). The simulation for the semiquinone signal was approximated as an S = 1/2 isotropic species (*g* = 2.003) without any hyperfine parameters included (Figure 2D). At 25 K, a single Fe/S signal was observed, allowing for the straightforward assignment of the remaining cluster in the composite spectrum at 10 K (Figure S3). Simulation of the experimental spectrum at 10 K yielded *g*-values of *g*_1,2,3_ = 2.075, 1.926, 1.855 for [4Fe-4S]-I (seen at 10 K) and *g*_1,2,3_ = 2.047, 1.944, and 1.873 for [4Fe-4S]-II (seen at 10 and 25 K) with integrated spin intensities of approximately 0.8:1 (Figures 2B,2C and S3). As described below, we are unable, at present, to directly assign these signals to the specific clusters of EtfX (Figure 1). Comparing the signals from the two [4Fe-4S] clusters, it should be noted that the significantly broader nature of the signal from [4Fe-4S]-I likely underestimates its contribution to the composite spectrum, and this is also the case for the variants described below. Thus, despite the calculated ratios presented herein, both clusters are likely fully reduced and present in comparable amounts.

**Figure 3:**
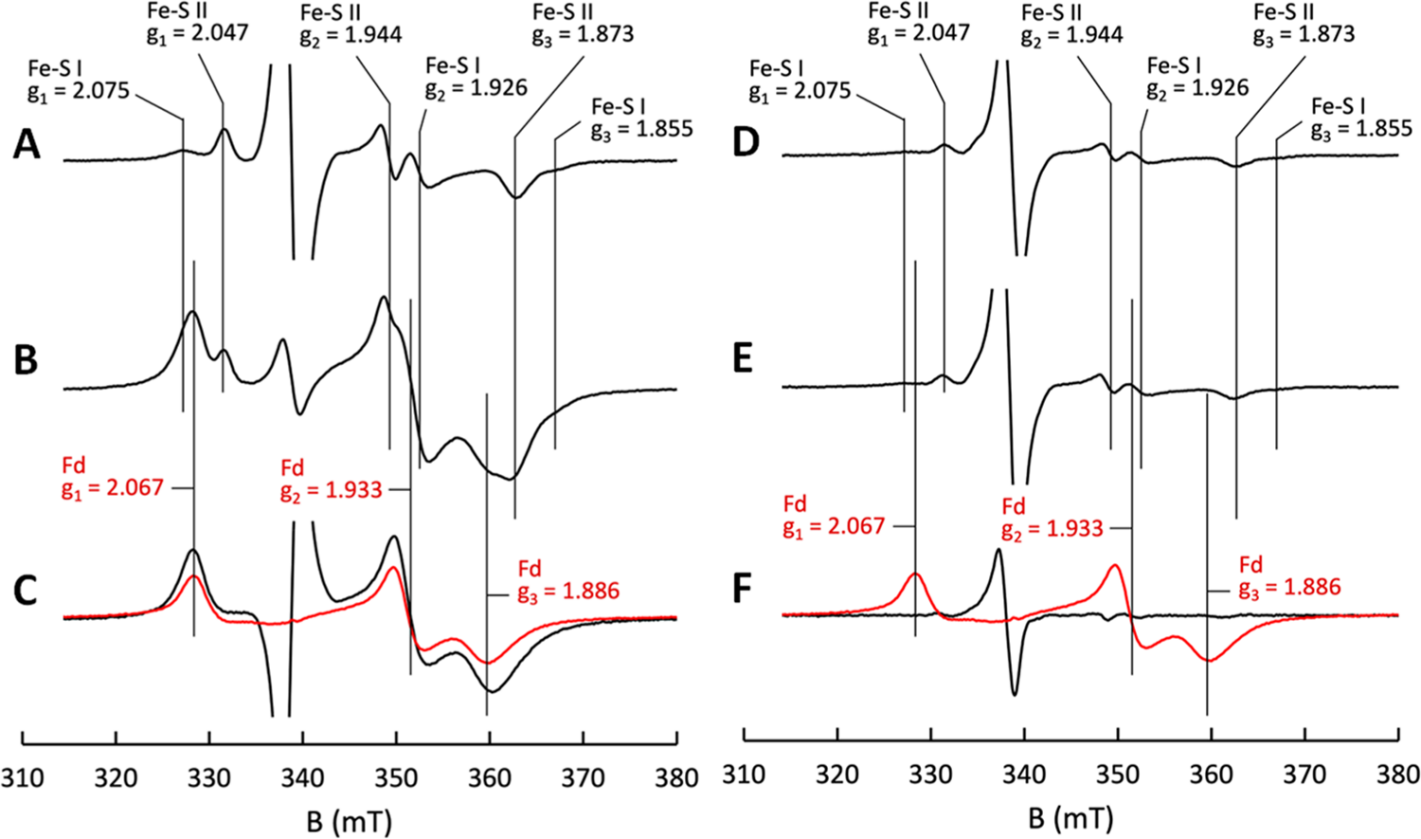
Estimate of the extent of ferredoxin reduction with the EtfABCX wild-type and the R140Q mutant. EtfABCX Wild-type: (A) EPR spectrum seen with 23.1 μM wild-type EtfABCX reduced by 480 μM NADH. The large isotropic signal in the middle of the spectrum is due to the presence of flavin semiquinone in EtfABCX. (B) Spectrum seen with 23.1 μM EtfABCX and 480 μM NADH in the presence of 120 μM ferredoxin. (C) Spectrum of a 21.4 μM reduced ferredoxin standard (red) overlapped with the difference spectrum of (B) minus (A) (black). EtfABCX R140Q: (D) EPR spectrum seen with 23.1 μM R140Q EtfABCX reduced by 480 μM NADH. The large isotropic signal in the middle of the spectrum is due to the presence of flavin semiquinone in EtfABCX R140Q. (E) Spectrum seen with 23.1 μM EtfABCX R140Q and 480 μM NADH in the presence of 120 μM ferredoxin. (F) Spectrum of a 21.4 μM reduced ferredoxin standard (red) overlapped with the difference spectrum of (E) minus (D) (black). The large inverted derivative feature in the difference spectrum is due to variable amounts of the semiquinone in the two samples. The amount of ferredoxin reduced was estimated by taking the ratio of the *g*_1_ values from the reduced ferredoxin standard and the difference spectrum. The samples were prepared in 50 mM HEPES, 200 mM NaCl, pH 7.5 at 25 °C. The amount of ferredoxin reduced is presented in Table 1.

EPR spectroscopy was also used to investigate the electron bifurcation activity of EtfABCX using *T. maritima* ferredoxin as the low-potential acceptor, NADH as the midpotential donor, and the endogenous menaquinone bound to the purified holoenzyme as the high-potential acceptor. *T. maritima* ferredoxin contains a single [4Fe-4S] cluster,^34^ and the dithionite-reduced recombinant protein gave rise to a well-defined rhombic EPR signal with *g*_1,2,3_ of 2.067, 1.933, and 1.886 (Figure 3C, red spectrum), in good agreement with the values (*g*_1,2,3_ of 2.06, 1.93, and 1.89) previously determined.^20^

Addition of both ferredoxin (120 μM) and NADH (480 μM) to EtfABCX (23.1 μM) gives rise to the EPR absorption shown in Figure 3B. The spectrum consists of four overlapping signals from the reduced forms of [4Fe-4S]-I and [4Fe-4S]-II, the flavin semiquinone, and reduced ferredoxin. The amount of ferredoxin reduction can be estimated by subtracting the contributions from the reduced [4Fe-4S] clusters of EtfABCX from the composite spectrum and comparing the resulting spectrum with that observed from the reduced ferredoxin standard using the g_1_ maximum for direct comparison (Figure 3). The results indicate that approximately 32 μM ferredoxin becomes reduced, which corresponds to approximately 1.4 equiv per EtfABCX present (23.1 μM) (Table 1). Hence, the low-potential ferredoxin is reduced by EtfABCX using NADH as the electron donor and endogenous menaquinone as the high-potential electron acceptor. The stoichiometry indicates that 1.4 mol of NADH is oxidized, which results in the reduction of 1.4 mol of ferredoxin (a one-electron acceptor), and 0.7 mol of menaquinone (a two-electron acceptor), assuming that quinone is present in stoichiometric amounts (one menaquinone per EtfABCX).

**Table 1:** Estimation of the Extent of Ferredoxin Reduction

sample^a^	[Fd_red_]^b^ (μM)^b^	[Fdred]/[EtfABCX]
WT	32	1.4
R140M	0	0
R140Q	0	0
R38Q	12.8	0.6
P239G	19.8	0.9^b^
V242G	13.7	0.6

a Samples contained 23.1 μM EtfABCX, 120 μM ferredoxin (Fd) and 480 μM NADH and were incubated at pH 7.5 and 25 °C.

b Values represent the average of duplicates.

### Role of Specific Amino Acid Residues on the Properties of BF-FAD in EtfABCX and EtfAB

To investigate the roles of amino acids in the vicinity of the BF-FAD of EtfABCX on its properties, a phylogenetic analysis was performed on *T. maritima* EtfAB. A total of 43 sequences were selected based on the five groups of electron-transferring flavoproteins identified by Costas et al.,^16^ with additional sequences not included in that study from proteins that have subsequently had their structures determined^16^ (Table S2A and S2B). These additional structures included the EtfAB subcomplexes from *T. maritima* (Tm_1530, PDB: 7KOE), CarDE (H6LGM7, PDB: 6FAH) from *Acetobacterium woodii*,^18^ and Fix/EtfAB (Q6N104) from *Rhodopseudomonas palustris*,^29^ all of which fall into the group 2 family of ETFs (Figures S4 and S5).

**Figure 4:**
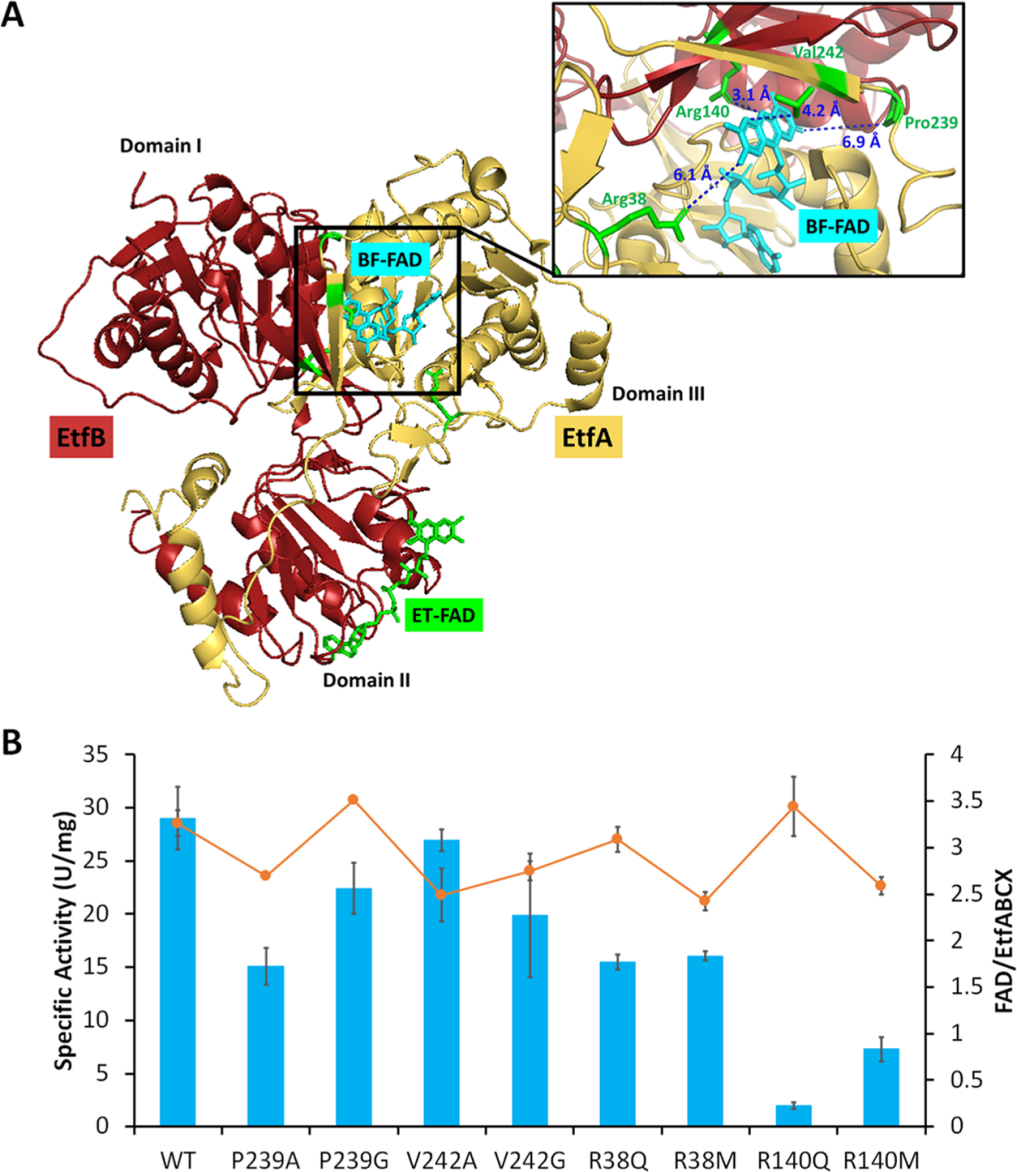
Site-specific mutants of EtfABCX and their NADH oxidation activities. (A) EtfA (yellow) and EtfB (red) structures from the EtfABCX Cryo-EM structure (PDB: 7KOE). BF-FAD and ET-FAD are colored cyan and green, respectively. Domains I and II in EtfB and domain III in EtfA are indicated in panel (A). Conserved residues Arg38, Val242, and Pro239 in EtfA and Arg140 in EtfB surrounding BF-FAD were labeled in green in the inset. Their distances to BF-FAD are labeled using blue dotted lines with distances of 6.1, 4.2, 6.9, and 3.1 Å, respectively. (B) Dye-linked NADH oxidation assay contained the wild-type and mutant forms of purified EtfABCX (15 μg) in 50 mM HEPES, pH 7.5, and 100 mM NaCl buffer with 0.5 mM NADH and 0.2 mM iodonitrotetrazolium chloride in anaerobic cuvettes. Reactions were started by injecting NADH. Specific activities are expressed in U/mg where 1 U represents 1 μmol of INT reduced per minute (blue bars). The orange curve indicates FAD occupancy (moles per mole of EtfABCX) in each protein sample.

The cryo-EM structure of *T. maritima* EtfABCX^9^ revealed that there are several amino acids in the vicinity of the BF-FAD that could play pivotal roles in determining the properties of this unique flavin and impact electron bifurcation and/or the transfer of electrons from NADH oxidation. Specifically, as shown in Figure 4, the EtfA residues Arg38, Pro239, and Val242 are 6.1, 6.9, and 4.2 Å away from the BF-FAD, respectively, while Arg140 of EtfB is at a distance of 3.1 Å. EtfA-Arg38 and EtfA-Val242 make van der Waals contacts with the flavin ring of BF-FAD, while EtfA-Pro239 causes a bend in the protein backbone to the BF-FAD based on the Cryo-EM structure.^9^ EtfA-Arg38, Val242, and Pro239 are all conserved residues in group 2 sequences; EtfA-Arg38 belongs to a conserved motif RXGV in most group 2 sequences, and Val242 and Pro239 belong to another conserved motif (GXXSPTXV) in an outer loop of EtfA that wraps around the BF-FAD and also extends into the ET-FAD domain (Figure S5A). EtfB-Arg140 is only 3.1 Å from BF-FAD-N(5) and forms hydrogen bonds with BF-FAD. EtfB-Arg140 belongs to a conserved motif RPXFGG in group 2, but R140 itself was also conserved in some other sequences that belong to proteins in all five groups (Figure S5B).

To determine these residues’ roles in electron bifurcation and transfer, each was mutated individually to one of two residues, and all eight EtfABCX variants were recombinantly expressed in *E. coli* and purified. The variants were V242A/G, R38Q/M, and P239A/G in EtfA, and R140Q/M in EtfB (henceforth, we will just refer to each variant without reference to the subunit; Table S1). Fortuitously, each of the variants also yielded the corresponding EtfAB subcomplex, in proportions similar to those observed with the holoenzyme to those observed with the wild-type enzyme (Figure S1). Flavin is not bound as tightly to the EtfABCX and EtfAB variants as compared with WT, even with FAD added to the buffers used during purification. While the WT EtfABCX and EtfAB contain close to three and two FAD molecules, respectively (Table S3), the EtfAB variants typically contained lower flavin content (from 44.1% to 73.7% of the expected two) than their corresponding EtfABCX variants (ranging from 74.3% to 107.5% of the expected three; see Table S3). This is not unexpected since the Cryo-EM structure shows the BF-FAD is in a relatively stable pocket in EtfA domain III, while the ET-FAD is located in a pocket that is made by EtfB domain II and EtfX (Figure 4A).^9^ Obviously, the EtfAB subcomplex lacks EtfX, so the binding affinity of ET-FAD will be lower than it is in the holoenzyme. Hence, the EtfAB variants typically contained the BF-FAD, indicating that mutation of the conserved residues, Pro239, Val242, Arg38 and Arg140, did not compromise its binding. This was confirmed by NADH titrations, as discussed below, which demonstrated the ability of NADH to reduce the variants. Interestingly, the glycine variants of both P239 and V242 showed slightly higher FAD content (3.51 and 2.75, respectively) than the corresponding alanine variants (2.69 and 2.49, respectively, Table S3). Meanwhile, the two arginine variants (Arg38 and Arg140) both showed higher flavin content when replaced by glutamine rather than methionine (3.09 vs 2.42 in Arg38 and 3.44 vs 2.59 in Arg140; Table S3). The five EtfABCX variants P239G, V242G, R38Q, R140M, and R140Q that contained approximately three flavins per holoenzyme were chosen for further spectroscopic characterization for comparison to WT EtfABCX.

### Catalytic Activities of EtfABCX and EtfAB Variants

**Figure 5:**
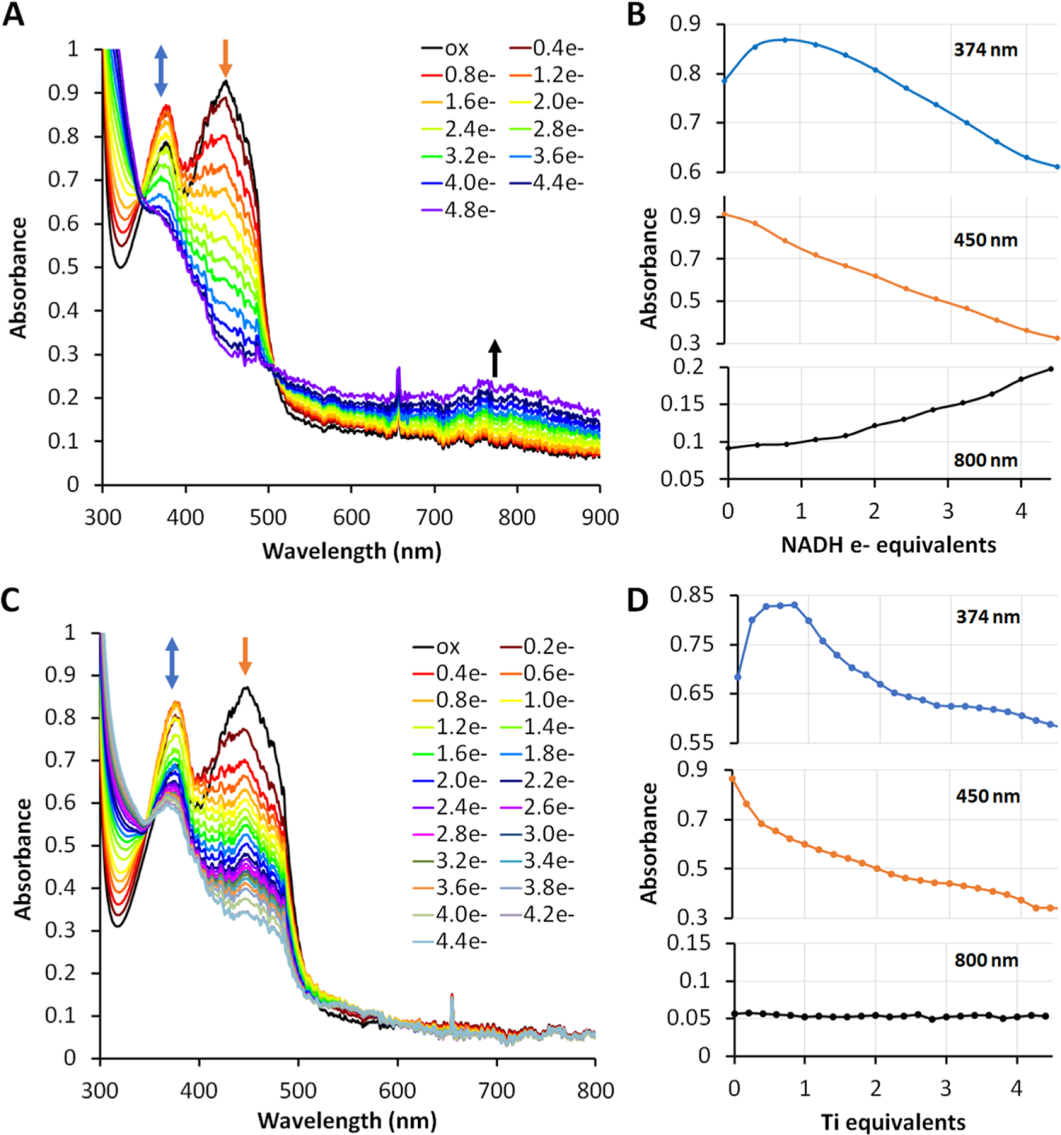
Anaerobic titration of WT EtfAB with NADH (A, B) and Ti (III) (C, D). WT EtfAB (50 μM) contained 1.79 ± 0.09 atoms of FAD per complex. Samples were air-oxidized and then titrated with NADH or Ti (III) in anaerobic cuvettes. Blue arrows indicate formation and disappearance of the FAD^•–^, orange arrows indicate disappearance of OX flavin absorbance, and black arrows indicate the formation of a CT complex in panels (A) and (C). (B) Absorbance change of flavin at 374 nm (blue), at 450 nm (orange), and 800 nm (black) when titrated with NADH. (D) Absorbance change of flavin at 374 nm (blue), at 450 nm (orange), and 800 nm (black) when titrated with Ti.

The specific activity for NADH oxidation in the dye-linked assay of wild-type EtfAB (13.7 U/mg) was less than half that of the wild-type holoenzyme (30.1 U/mg), contrary to what would be expected, given the differences in their molecular weights (68 and 128 kDa, respectively, Figures 4B and S6). Hence, the NADH-to-dye turnover number for EtfAB is only about 20% that of EtfABCX. All of the EtfAB variants analyzed contained approximately one FAD (Table S3) and P239G, V242G, and R38Q exhibited approximately 50% of the NADH oxidation activity of wild-type EtfAB, while R140Q and R140M were virtually inactive (Figure S6). This suggests that the single FAD in these EtfAB variants is the BF-FAD as ET-FAD is not thought to be able to directly interact with NADH. This was confirmed by anaerobic NADH titration of P239G, V242G, and R38Q variants of EtfAB (Figure S7). For the wild-type EtfAB, the FAD absorption feature at 450 nm decreased until 4 electron equivalents of NADH had been added (Figure 5A,B), but only 2.8, 1.6, and 1.6 equiv could be added to P239G, V242G, and R38Q variants, respectively (Figure S7), consistent with their FAD contents (Table S3) and the activity assays (Figure S6). However, the Arg140 variants (R140Q/M) lost virtually all NADH oxidation dye-linked activity, even though they still have about one FAD present in the EtfAB subcomplexes and can be reduced by NADH in titration (Figures S6 and S7). Hence, the single flavin in the R140Q/M variants is BF-FAD, and Arg140 appears to play an important role in its initial reduction by NADH and also the subsequent transfer of reducing equivalents out of the BF-FAD.

For the EtfABCX variants, the P239 and R38 variants showed dye-linked NADH oxidation activities lower than those of the wild-type enzyme (Figure 4B). Decreased activity might be due to structural changes in the P239G and R38Q complexes as their FAD contents are close to three per mole (Table S3). Once more, the Arg140 variants are unique as these showed much lower activities than the other variants even though they contained three FAD/holoenzyme. Arg140 is clearly crucial for the electron transfer from NADH in the dye-linked assay. The EPR of the two [4Fe-4S] clusters in the dithionite-reduced R140Q and R140M EtfABCX variants were indistinguishable from those of the wild-type enzyme (Figures 2 and S8). On the other hand, neither R140 variant was able to reduce ferredoxin. Obtaining the difference spectra between samples with and without ferredoxin, as described above, showed no evidence of ferredoxin reduction (Figures 3F and S9C). Additionally, the level of reduction of the iron–sulfur clusters in both of these variants was considerably lower than that seen with the wild-type EtfABCX (compare Figures 3A–3D and S9A). It thus appears that the Arg140 residue is essential for electron bifurcation activity.

The R38Q, P239G, and V242G variants of EtfABCX all contained approximately three FAD/mol (Table S3) but exhibited only about 50–70% of the NADH oxidation activity of the wild-type (Figure 4B). The dithionite-reduced variant enzymes exhibited EPR signals characteristic of the two [4Fe-4S] clusters that were indistinguishable from the wild-type complex. For R38Q, the EPR signals for the two clusters were in a 0.8:1 ratio (Figure S10), and for P239G, the EPR signals of the two clusters were in a 1:1 ratio (Figure S12), while with V242G the ratio was 0.7:1 with a lower EPR absorption for the [4Fe-4S]-I cluster (Figure S14). However, the iron contents of the three samples were not significantly different (Table S4), suggesting that both clusters are fully occupied. Moreover, it is not likely that the [4Fe-4S]-I cluster in the V242G variant has a significantly lower reduction potential than in the wild-type enzyme. In contrast to the Arg140 variants, the R38Q, P239G, and V242G variants of EtfABCX did exhibit NADH- and ferredoxin-dependent electron bifurcation activity (Figures S11, S13, and S15), although the ability of R38Q, P239G, and V242G variants to reduce ferredoxin was diminished to 0.6, 0.9, and 0.6 ferredoxin, respectively, compared to 1.4 for the wild-type enzyme (Table 1).

### Reductive Titrations of WT and Arg140 Variants of EtfAB and EtfABCX

The UV–vis spectrum of as-purified EtfAB indicated that one of the flavins was in the FAD^•–^ state (Figure S16A). This was previously observed with as-purified EtfABCX from *P. aerophilum* and was assigned to the ET-FAD.^8^*T. maritima* EtfAB was oxidized by brief air exposure and the fully oxidized enzyme was titrated with NADH anaerobically (Figure 5). This also generated an intermediate FAD^•–^ species as reflected by the absorbance increase at 374 nm, which reached a maximum when approximately half an equivalent of NADH had been added. The absorbance at 374 nm then decreased with further addition of NADH until both flavins were fully reduced, with no further decrease in the absorbance at 450 nm (Figure 5A,5B). Thus, oxidation of one NADH generates the singly reduced FAD^•–^ forms of BF-FAD and ET-FAD, as observed in the as-purified subcomplex (**S16A**). Similar FAD^•–^ states were observed previously in the course of NADH titration of EtfAB-Bcd of *Acidaminococcus fermentans* and EtfAB of *P. aerophilum*.^8,22^ In addition, a charge-transfer (CT) band was seen from 550 to 900 nm (Figure 5A,5B), likely due to the interaction of NAD^+^ with the fully reduced BF-FAD.^16^ This assignment was supported by titration of EtfAB with the chemical reductant titanium(III) citrate (Ti). This also generated the FAD^•–^ signature at 374 nm reaching a maximum with one-electron equivalent, while the absorbance at 450 nm reached a minimum when four equiv were added (Figure 5C). However, the charge-transfer band (700–900 nm) was not observed until NAD^+^ was added (Figures 5D and S17). The Arg140 variants (R140Q and R140M) of EtfAB contained approximately one FAD/mol (Table S3) and had no dye-linked NADH oxidation activity (Figure S6) but still showed decreasing absorption at 450 nm when titrated by NADH. Interestingly, the EtfAB R140Q or R140M variants appear to remain partially reduced as the FAD^•-^ state of the single FAD after being exposed to air for hours before being titrated by NADH (Figure S7D and S7E) but there was no evidence of a flavin semiquinone by EPR analysis (data not shown).

**Figure 6:**
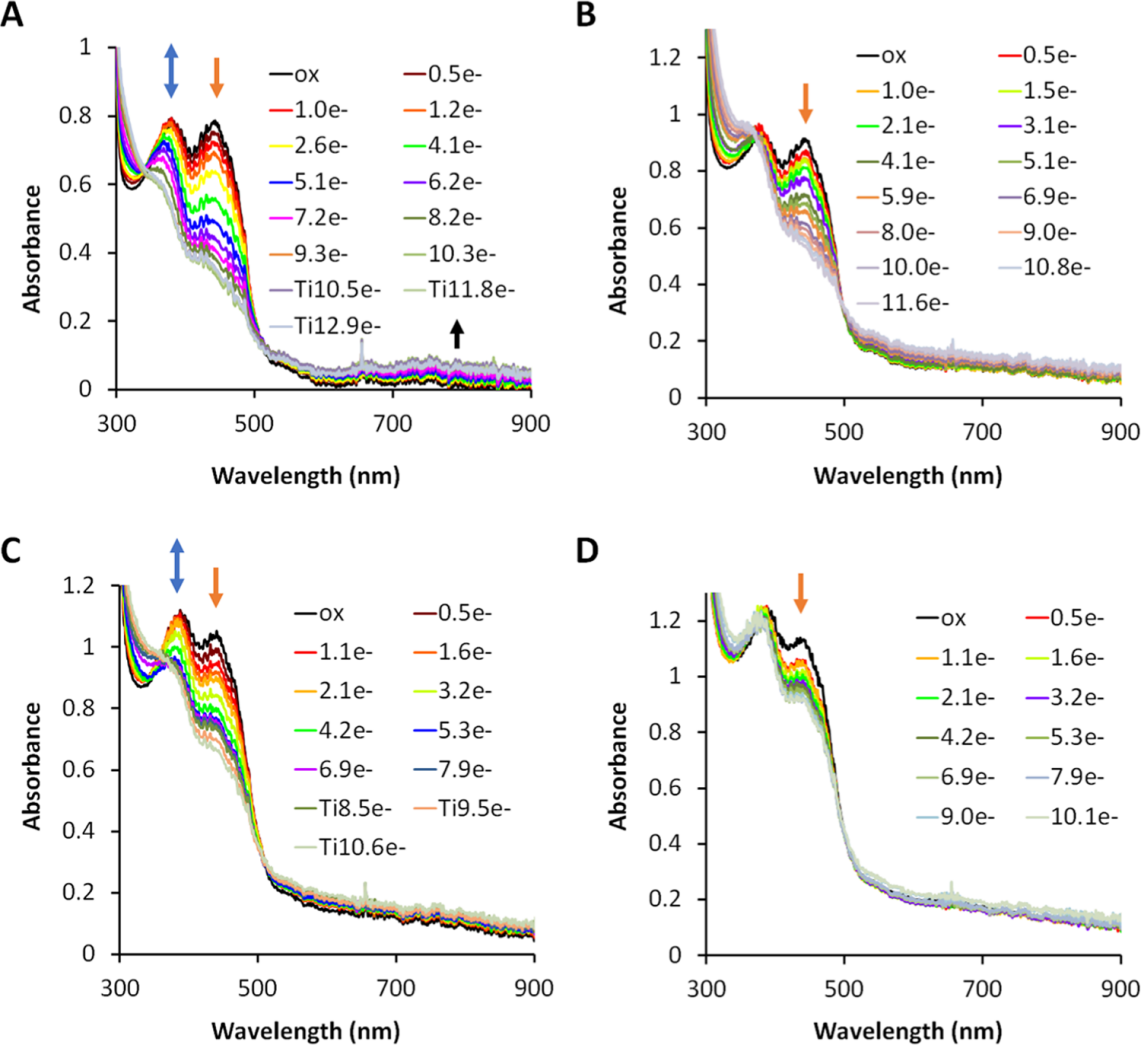
Anaerobic titrations of the EtfABCX WT and R140Q mutant with NADH and Ti(III) monitored by visible spectroscopy. 15 μM of air-oxidized EtfABCX WT with 3.26 ± 0.14 of FAD was titrated by NADH to 10 e– equivalents and then further titrated by Ti(III) to 13 e– equivalents in panel (A) or was titrated by Ti(III) in panel (B). 15 μM of air-oxidized EtfABCX R140Q with 3.44 ± 0.32 of FAD was titrated by NADH to 8 e– equivalents and then further titrated by Ti(III) to 11 e– equivalents in panel (C) or was titrated by Ti(III) in panel (D). Blue, orange, and black arrows indicate absorbance changes at 374, 450, and 800 nm, respectively.

**Figure 7:**
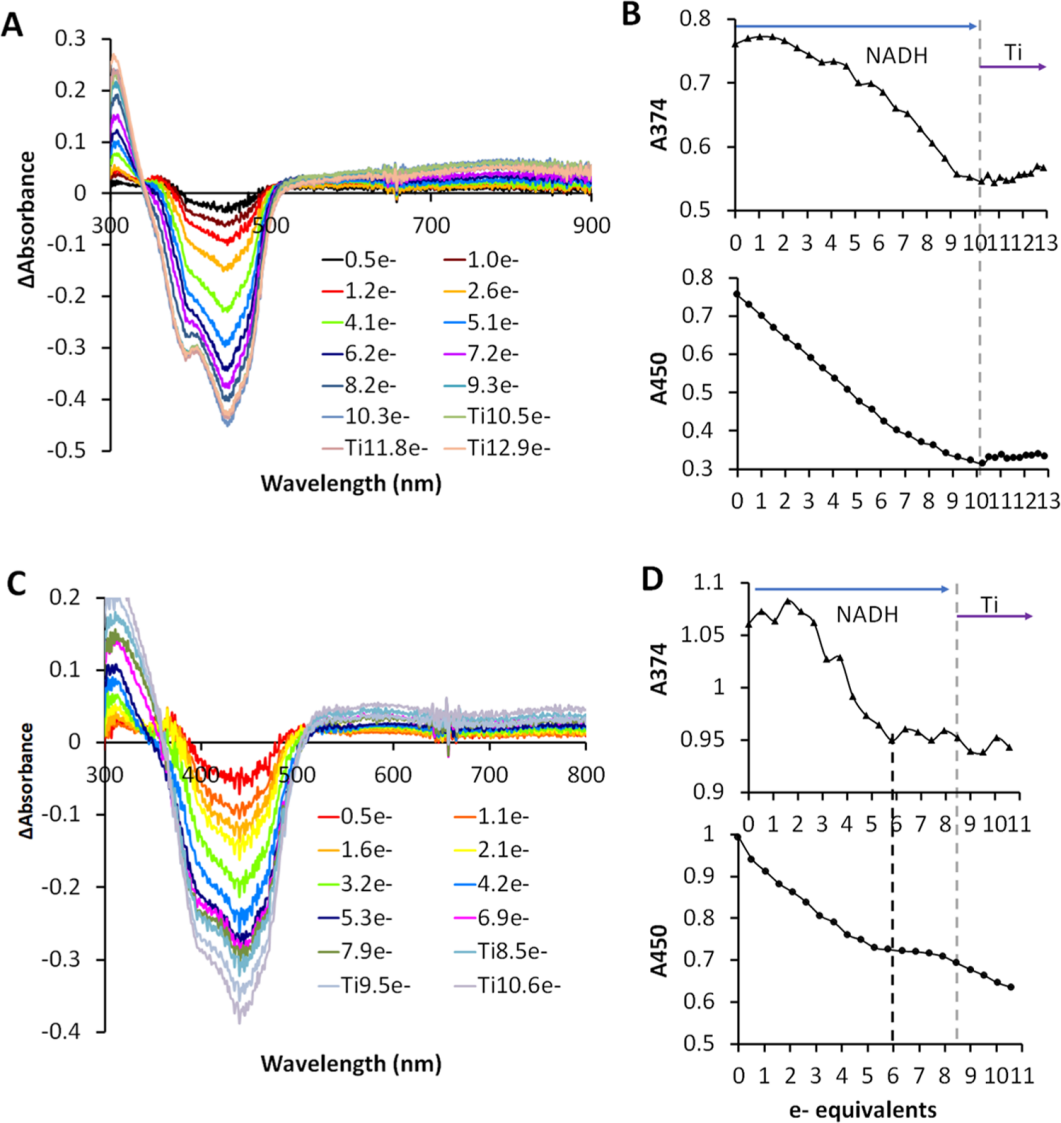
Anaerobic titrations of the EtfABCX WT and R140Q mutant with NADH and Ti(III). Difference spectra of EtfABCX WT (A) and R140Q (C) derived from the data in Figure 6. Absorbance changes at 374 and 450 nm for EtfABCX WT (B) and R140Q (D) titrations. Blue arrows indicate NADH titration and purple arrows indicate Ti(III) titration after NADH in the same cuvettes. Black dashed lines were used to indicate four-electron equivalents in R140Q titration and gray dashed lines were used to indicate the end of NADH titration and the beginning of Ti(III) titration.

The UV–vis spectrum of the as-purified full EtfABCX complex also had the ET-FAD in the FAD^•–^ state (Figure S16B). When wild-type EtfABCX was fully oxidized by air and then titrated (anaerobically) with NADH (Figure 6A), approximately five equiv of NADH were oxidized based on absorbance changes at 450 nm. The 10 electron equivalents are assumed to reduce not only the three flavins (BF-, ET-, and QR-FAD, two for each), but also to reduce the two [4Fe-4S] clusters (by up to one electron each) and menaquinone (by up to two electrons) (Figure 7A,7B). Similar results were seen with Ti (III) indicating nearly complete reduction of the holoenzyme by NADH (Figure 6B).

In contrast to the wild-type enzyme, the R140Q variant of EtfABCX could only be reduced by six electrons using NADH as the reductant, although Ti (III) resulted in a further decrease in the absorption at 450 nm without any increase in absorbance at 374 nm, indicating that an additional flavin is reduced (Figures 6C and 7C,7D). This suggests that NADH can only partially reduce some of the redox-active cofactors in the enzyme via BF-FAD, distributing them among ET-FAD, QR-FAD, the two [4Fe-4S] clusters, and menaquinone, while reduction is much more complete with the nonspecific chemical reductant Ti (III).

During titration with NADH, formation of the FAD^•–^ was observed in WT EtfABCX and in the Arg140Q, as indicated by a shoulder near 374 nm in the difference spectrum (Figure 7A,7C). However, the shoulder was not as intense as in the WT EtfAB subcomplex (Figure 5), which probably results from the spectral interference from the two [4Fe-4S] clusters and QR-FAD. An interesting observation is that a charge-transfer band (600–900 nm) was observed in the NADH titration of EtfABCX WT but not in either of the two Arg140 variants (Figures 6C and S18A). The EtfABCX R140M NADH/Ti(III) titrations showed trends very similar to those of the R140Q NADH titrations (Figure S18). Clearly, Arg140 plays a crucial role in electron transfer during electron bifurcation by EtfABCX.

We attempted to investigate the role of the two [4Fe-4S] clusters in electron bifurcation by mutating the Cys coordinating either the proximal or the distal [4Fe-4S] in EtfX. These were the C29A and C29A/C33A single and double variants in EtfX for the distal [4Fe-4S], and the C61A and C61A/C64A single and double variants in EtfX for the proximal [4Fe-4S]. We were only able to obtain intact EtfABCX complex for the EtfX C29A variant, although all generated EtfAB forms. Presumably, the mutations led to incomplete incorporation of the [4Fe-4S] clusters and an unfolded EtfX that prevented the assembly of the holoenzyme. The exception, EtfABCX C29A, contained approximately 3 Fe atoms per mole (Table S4), suggesting that EtfX lacked the distal [4Fe-4S] cluster but still contained the proximal one, albeit in less than a stoichiometric amount. However, EPR spectroscopy showed that this was not the case. The dithionite-reduced variant enzyme gave rise to the complex EPR signal seen from the native enzyme characteristic of two clusters in a 1:1 ratio (data not shown). Hence, partial non-Cys ligation did not significantly affect the EPR properties of the distal [4Fe-4S] and approximately 40% of the EtfABCX C29A variant contained both clusters, while 60% lacked both clusters and little if any of the protein contained a single [4Fe-4S] cluster. Both [4Fe-4S] clusters, therefore, need to be present to stabilize the holoenzyme.

## Discussion

To date, the mechanism of electron bifurcation in Etf-type complexes remains poorly understood. The recombinant expression of wild-type and selected mutants of EtfAB-type holoenzymes is critically important to elucidate the molecular basis of bifurcation in this large class of bifurcating flavoproteins. Only three studies have been reported on the mutagenesis of EtfAB systems^18,24,35^ and these have included variants of the analogous R140 position in the *T. maritima* enzyme identified here as being critical in imparting bifurcating activity to the system. The work of Miller et al. suggested the importance of this conserved Arg (in this case, R165), which lies in close proximity to the BF-FAD in the EtfAB subcomplex from *R. palustris* as its mutation resulted in loss of the BF-FAD in the subcomplex.^35^ However, the *R. palustris* holoenzyme was not studied, and FAD might possibly be more stable in the Arg variant of the heterotetrameric enzyme. On the other hand, mutagenesis of the bifurcating caffeoyl-CoA reductase (CarCDE) from *A. woodii* by Demmer et al.^18^ showed that mutation of the homologous conserved R203 abolishes FAD-linked bifurcating activity with caffeoyl-CoA, while the dye-linked activity with NADH was almost an order of magnitude lower. However, the ferredoxin-linked bifurcating activity was not determined for the mutants, and no spectroscopic characterization was carried out. More recently, in the structural and mutational analyses of the bifurcating Ldh-EtfAB from *A. woodii*, when the arginine interacting with the BF-FAD (Arg205) was mutated to alanine, there was a complete loss of ferredoxin-linked bifurcating activity, with retention of full NADH-to-dye activity.^24^ Although each is incomplete in itself, these studies suggest that this conserved Arg residue plays a critical role in imparting bifurcating activity in all of these enzymes.

Herein, we have utilized EPR spectroscopy to ascertain the reduction of iron–sulfur clusters in both the holoenzyme and ferredoxin to assess the bifurcation activity in the wild-type complex and in the mutants in the immediate vicinity of the BF-FAD. To achieve this, we have successfully recombinantly expressed in *E. coli* active forms of the EtfAB subcomplex and the EtfABCX holoenzyme of *T. maritima* and examined them by both EPR and UV/vis spectroscopy. The wild-type holoenzyme exhibits electron bifurcation activity as measured by its ability to reduce ferredoxin. This is in the absence of added water-insoluble menaquinone and, instead, relies on the endogenous menaquinone that was bound to the enzyme. Based on our previous structural analysis, the EtfABCX complex incorporates quinone during anaerobic expression in *E. coli*, which produces menaquinone when grown in the absence of oxygen.^9,36^

In the present work, we have targeted four conserved amino acids in the vicinity of the BF-FAD in the Group 2 family of Etf for mutagenesis, P239, V242, and R38 in the EtfA subunit, and R140 in the EtfB subunit (Figure S5). The R140 residue with a p*K*_a_ of ∼12 is protonated and positively charged at neutral pH and belongs to the conserved motif **R**PXFGG in Group 2 sequences (Figure S5B). This has been mutated to either the polar glutamine and the hydrophobic methionine, both of which render the enzyme unable to reduce ferredoxin, as shown by our EPR spectroscopy. The absence of ferredoxin reduction with the R140Q and R140M variants clearly demonstrates that this residue is indeed critical in maintaining the environment around the BF-FAD necessary for bifurcation, likely due to the insufficiently low potential of BF-FAD^•–^ such that it cannot reduce ferredoxin. This is also suggested by a computational study indicating that the conserved arginine that hydrogen bonds to the N5 of the BF-FAD might be important to maintain the hydroquinone state of BF-FAD.^37^ At the same time, the present work indicates that reduction of the other redox-active centers in EtfABCX is also affected upon reduction of the complex by NADH, even though all can be fully reduced by the chemical reductant dithionite. In particular, our EPR analysis reveals that the two [4Fe-4S] clusters of EtfX are not significantly reduced by NADH (Figure 3D), again reflecting the loss of bifurcating activity. Hence, in the absence of R140, the BF-FAD cannot transfer electrons to the high-potential pathway to ultimately reduce quinone as well as to the low-potential pathway to reduce ferredoxin.

By contrast to R140 in EtfB, the present work shows that the conserved R38, P239, and V242 residues were not as significant in imparting bifurcating activity as variants with mutations at each position retain at least partial bifurcation activity as detected by EPR-monitored reduction of ferredoxin. Both motifs might, nevertheless, play important roles in maintaining the conformational structure; for example, the GXXSPTXV motif is located in a hinge region that extends into EtfB Domain II (Figure 4A). Consistent with this, we find that mutation of each of these residues appears not to bind ET-FAD as tightly as does wild-type EtfAB (Table S3). These EtfAB subcomplex variants had less than one FAD but were all active in the dye-linked assay and therefore must contain the BF-FAD, indicating that the missing flavin is the ET-FAD (Figure S6). This is supported by the cryo-EM structure in which the ET-FAD is found to be quite solvent-exposed in the absence of EtfX (Figure 4).^9^ This in turn suggests that alterations in the environment of the BF-FAD can also affect the binding of the ET-FAD. Hence, we can rule out the possibility that the lack of dye-linked NADH oxidation activity by EtfAB_R140Q and R140M (Figure S6), which also contained just one FAD, is due to the loss of BF-FAD as a result of the Arg140 mutation. Both R140Q and R140M variants of EtfAB, replete with both ET-FADs, remain in a partially reduced state following reduction by NADH and air-reoxidation, indicating that electron transfer between the two flavins and the subsequent electron flow down the high-potential pathway has been compromised. This would explain why the EtfABCX_R140Q and _R140M variants are reduced by only 3 equiv of NADH, while the wild-type enzyme can be reduced by five (Figures 7 and S18A) and the [4Fe-4S] clusters in EtfX do not get fully reduced.

From our structural analysis of the *T. maritima* EtfABCX holoenzyme, we have proposed a mechanism of electron bifurcation that also incorporates findings on other EtfAB systems.^9^ In particular, the cryo-EM structure, surprisingly, clearly shows that the two [4Fe-4S] clusters in EtfX are part of the high-potential pathway to quinone rather than of the low-potential pathway to ferredoxin. A key element of flavin-based electron bifurcation is that the half-potentials of the BF-FAD are highly crossed, meaning the quinone/semiquinone couple has a potential much lower than that of the semiquinone/hydroquinone couple. Thus, the first electron out of the fully reduced BF-FAD has a high reduction potential and is transferred to the ET-FAD (which is in the one-electron-reduced state), leaving the remaining electron in the BF-FAD as semiquinone, which has a very low potential and is able to reduce ferredoxin in a way that is thermodynamically favorable. This was first demonstrated by a study of the unrelated bifurcating enzyme, the NADH-dependent reduced ferredoxin NADP^+^ oxidoreductase (NfnAB from *Pyrococcus furiosus*), where transient absorption spectroscopy revealed a BF-FAD semiquinone with a potential of −920 mV.^15^ Similarly, photoreduction of the BF-FAD in *R. palustris* EtfAB generated a low-potential BF-FAD semiquinone that reduced the low-potential dye benzyl viologen.^38^

The results presented herein suggest that R140 of EtfB plays a role in generating the highly crossed half-potentials of the BF-FAD and its removal most likely results in a loss of bifurcating activity because the low-potential semiquinone required for reduction of ferredoxin is no longer generated. Surprisingly, our results show that the R140 variants also exhibit significantly reduced electron transfer into the high-potential pathway, with all four sites (the ET-FAD and the two [4Fe-4S] clusters and the QR-FAD) not becoming fully reduced in either the R140M or R140Q variants. Furthermore, although the P239G and V242G variants retained some bifurcating activity, they also indicated that the mutations affected the affinity of EtfAB for the ET-FAD and interfered with efficient electron transfer between BF-FAD and ET-FAD as less ferredoxin was reduced compared to that seen with the wild-type enzyme.

Clearly, R140 is the key residue in determining the bifurcating ability of the BF-FAD of *T. maritima* EtfABCX. Miller and co-workers^37^ have suggested that upon reduction of the BF-FAD by NADH, the conserved Arg changes orientation and may be unable to contribute any electrostatic stabilization of the anionic semiquinone of the BF-FAD. This is now shown in a revised mechanism for the R140 variant where the enzyme becomes trapped and contains a BF-FAD^•–^ and oxidized [4Fe-4S] clusters in EtfX (Figure S19).

Interestingly, the conserved Arg140 next to BF-FAD found in EtfABX is not only found in EtfAB-type homologues, but homologous Arg residues are found next to the BF-FAD in two other phylogenetically unrelated bifurcating enzymes.^39^ In the NfnAB mentioned above (also from *T. maritima*), Arg187 of NfnB sits on top of BF-FAD-N5 (3.1 Å, PDB: 4YRY)^40^ and in hydrogenase/heterodisulfide reductase (Hdr-Mvh, from *Methanothermococcus thermolithotrophicus*), Lys409 (in HdrA) is very close to its BF-FAD-N(5) (3.4 Å, PDB: 5ODQ).^41^ The situation is quite different in the fourth major category of bifurcating enzymes, the Bfu family^5^ where the flavin (FMN) in these enzymes interacts with the high-potential acceptor substrate rather than midpotential electron donor and electron bifurcation is thought to be accomplished by a combination of FMN and at least two iron–sulfur clusters. In the *Acetomicrobium mobile* bifurcating NiFe hydrogenase (NiFe-BfuABCSL), no Arg (or Lys) is seen within 6 Å of FMN-N(5), suggesting a fundamentally different bifurcating mechanism.^42^ Nature thus appears to have evolved at least two ways to generate high-energy, low-potential electrons through electron bifurcation. While one remains to be elucidated, three of the four known enzyme families utilize FAD with an adjacent Arg residue playing a key and pivotal role in the bifurcating mechanism.

## Data Availability

Data not contained in the manuscript or Supporting Information (e.g., protein gels, vis absorption spectra, raw titration data, EPR spectra) are available on request to the corresponding authors (adamsm@uga.edu and russ.hille@ucr.edu).

## Supporting Information Available

Chromatographic separation of EtfABCX and EtfAB; temperature and pH dependence of activity; EPR spectra of the iron–sulfur clusters of EtfABCX; sequence alignments and phylogenetic analysis of EtfA and EtfB; nonbifurcating activities and FAD contents of EtfAB mutants; UV–vis spectra of NADH titrations of EtfAB; EPR spectra of R140M EtfABCX, R38Q EtfABCX, P239G EtfABCX, and V242G EtfABCX and their bifurcating activities; UV–vis spectra of EtfAB and EtfABCX; UV–vis spectra of NAD^+^ titrations of Ti-reduced EtfAB; UV–vis spectra of EtfABCX titrated with NADH and Ti; mechanism of electron bifurcation of EtfABCX and the impact of mutating R140M; nucleotide primers used in expressing *etfABCX*; and FAD and iron contents of mutants of EtfAB and EtfABCX (PDF)
Sequence details of the EtfA and EtfB homologue used in alignments and phylogenetic analysis (XLSX)

## Accession Codes

*T. maritima* Fix/EtfABCX, EtfA Tm1530 WP_004081902, EtfB Tm1531 WP_004081904, EtfC Tm1532 WP_004081907, EtfX Tm1533 WP_004081910.
