# Supplementary material for: Characterization of the Membrane-Associated Electron-Bifurcating Flavoenzyme EtfABCX from the Hyperthermophilic Bacterium Thermotoga maritima
Source: Biochemistry. 2023 Dec 7;62(24):3554–67. doi: 10.1021/acs.biochem.3c00473 (PMC10734219; doi:10.1021/acs.biochem.3c00473)
Supplement: Supplementary file 1 — bi3c00473_si_001.pdf [file bi3c00473_si_001.pdf]

Biochemistry

Characterization of the membrane-associated electron bifurcating flavoenzyme EtfABCX from the hyperthermophilic bacterium *Thermotoga maritima*

Xiaoxuan Ge[1], Gerrit J. Schut[1], Jessica Tran[2], Farris L. Poole II[1], Dimitri Niks[2],

Kevin Menjivar[2], Russ Hille[2] and Michael W. W. Adams[1*]

[1]Department of Biochemistry and Molecular Biology, University of Georgia, Athens, GA 30602, USA

[2]Department of Biochemistry, University of California, Riverside, Riverside, CA 92507, USA

*Correspondence to: Dr. Michael W. W. Adams: adamsm@uga.edu

Supplementary Information

Figures S1 – S19

Tables S1 – S4

Figure S1. Separation of recombinant EtfABCX and EtfAB by size exclusion chromatography and purification of Tma Fd. (A) Size exclusion column was used to separate recombinantly expressed EtfABCX and EtfAB with 9× His tag on EtfA subunit from HisTrap FF column. The earlier big peak, including the small shoulder, was EtfABCX complex while the late peak was EtfAB complex. The blue curve indicated protein absorbance at 280 nm, while the pink curve indicated FAD absorance at 450 nm. The bands of EtfA, EtfB, EtfC and EtfX were indicated by arrows on the SDS-PAGE gel picture on the right side of the picture. The size of each subunit in EtfABCX are indicated in the inset. (B) SDS-PAGE of purified Tma Fd.

A

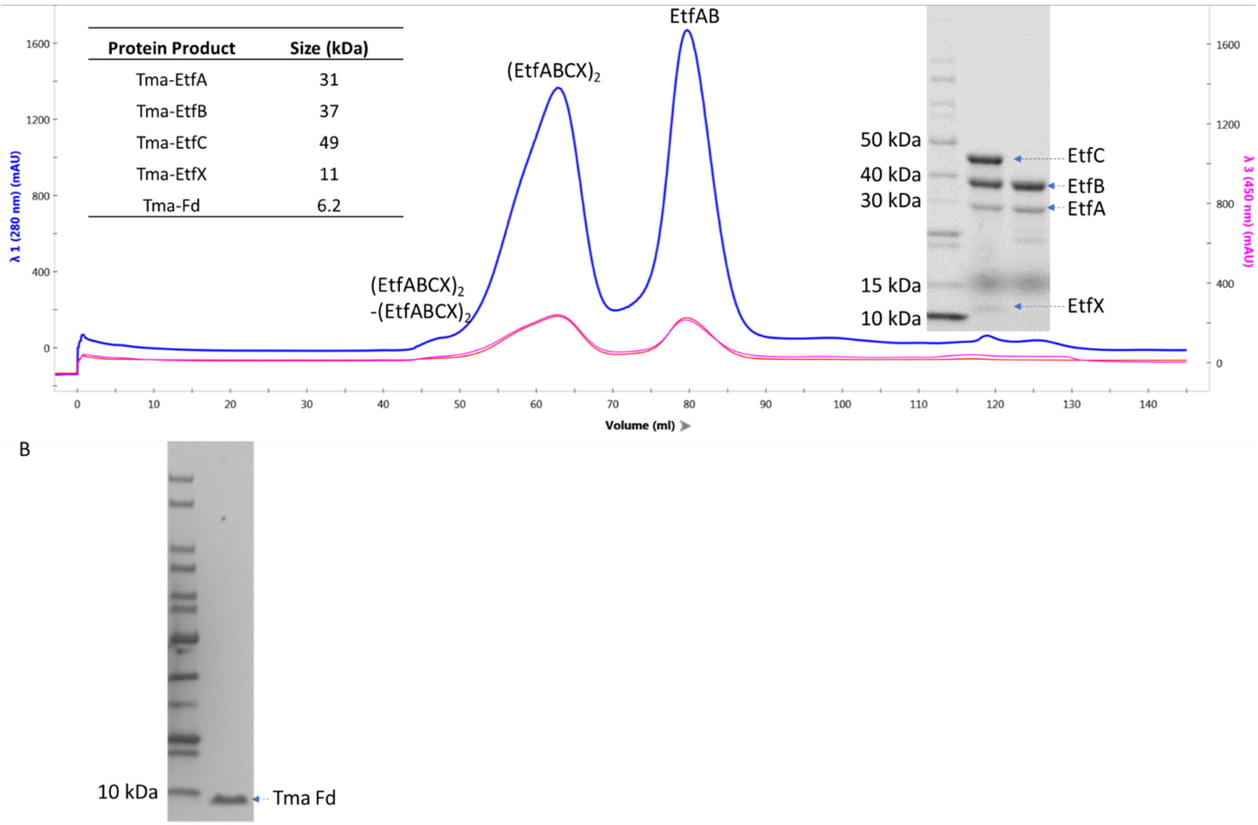

B

Figure S2. Temperature and pH dependence of the non-BF dye-linked NADH oxidation activity of EtfABCX. (A) The assay contained 15 µg purified EtfABCX in 50 mM HEPES, pH 7.5, 100 mM NaCl buffer with 0.5 mM NADH and 0.2 mM iodonitrotetrazolium chloride in anaerobic cuvettes. Temperature ranged from 25 °C to 85 °C. (B) The pH dependence of EtfABCX activity at 75 °C. This assay contained 15 µg purified EtfABCX in different buffers: 50 mM acetate, 100 mM NaCl for pH 5.0; 50 mM phosphate buffer, 100 mM NaCl for pH 6.0, 6.5, 7.0, 7.5 and 8.0; and 50 mM CHES, 100 mM NaCl for pH 9.0 and 10.0. Reactions were started by injecting NADH. Data were presented in percentage of maximum of dye-linked activity (U/mg, U represents µmoles of INT reduced per minute.)

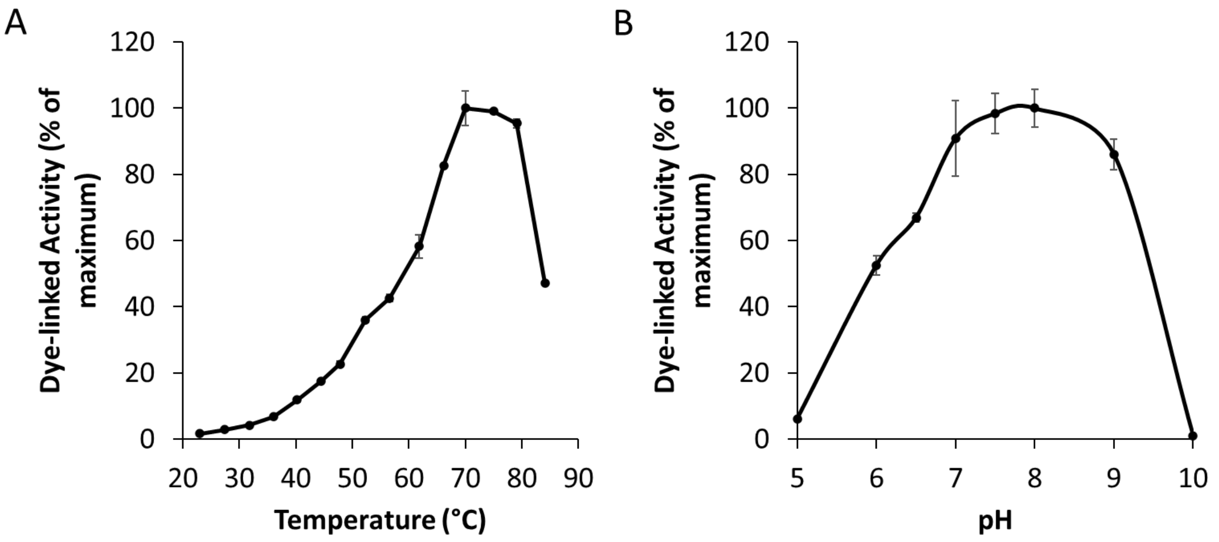

Figure S3. Temperature dependence of the EPR spectra of the iron-sulfur clusters of wild-type EtfABCX. (A) The spectrum collected at 10 K. (B) The spectrum collected at 25 K with 1 mT modulation amplitude and 8 mW power, exhibiting only one of the [4Fe-4S] clusters of EtfABCX and a small amount of flavin semiquinone. The reaction buffer used was 50 mM HEPES, 200 mM NaCl, pH 7.5.

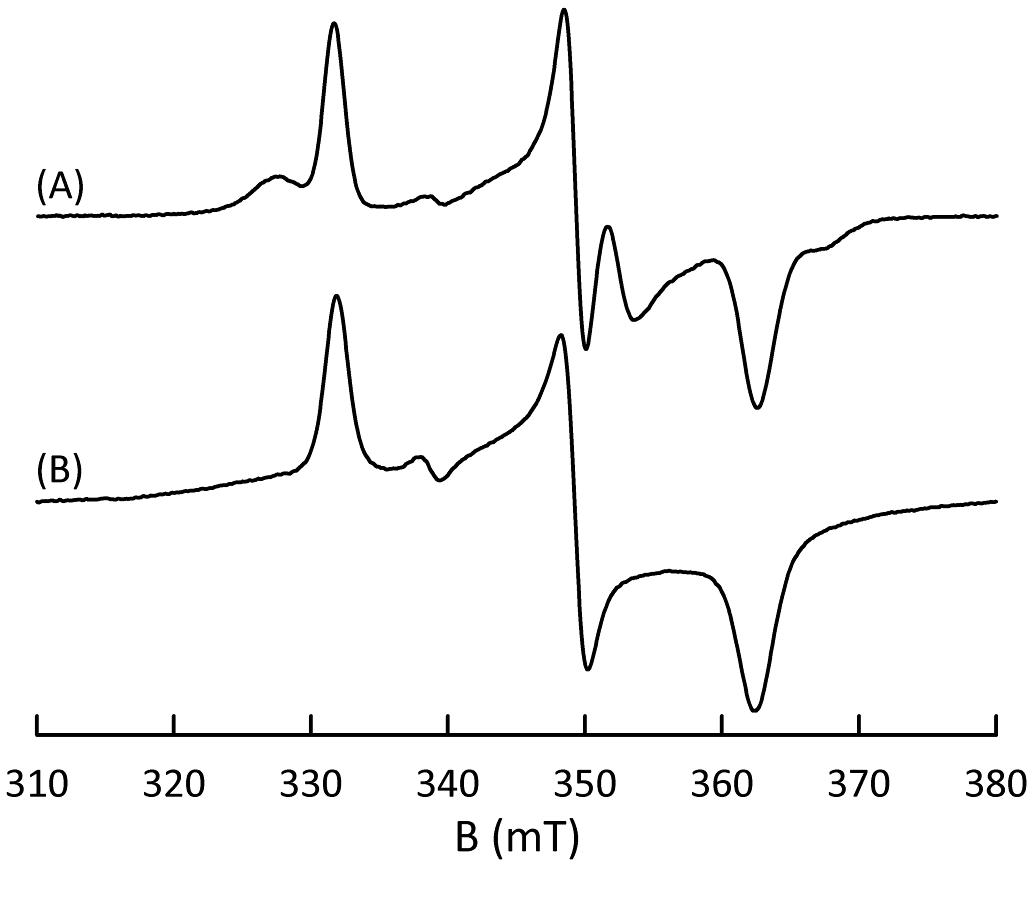

Figure S4. Phylogenetic analysis of *T. maritima* EtfA and EtfB with their homologs. Selected homolog sequences of *T. maritima* EtfA homologs (beta subunits) and EtfB homologs (alpha subunits) were first aligned individually, then the two resultant alignment blocks were concatenated following the names of the beta subunit sequences. Consensus phylogenetic tree was built here using Bootstrap as resampling method and the number of replicates was 100. Subunit sequences that were not analyzed in [16] (black triangle) were assigned to five corresponding groups in this analysis. Subunits with resolved structures were labeled by black stars. *T. maritima* EtfAB (TM_1530, PDB: 7KOE) was labeled by black square in this figure. CarDE (H6LGM7, PDB: 6FAH) from *Acetobacterium woodii* and Fix/EtfAB (Q6N104) from *Rhodopseudomonas palustris* were categorized in group 2. Details of sequences can be found in **Supplementary Table S2A** and **S2B**.

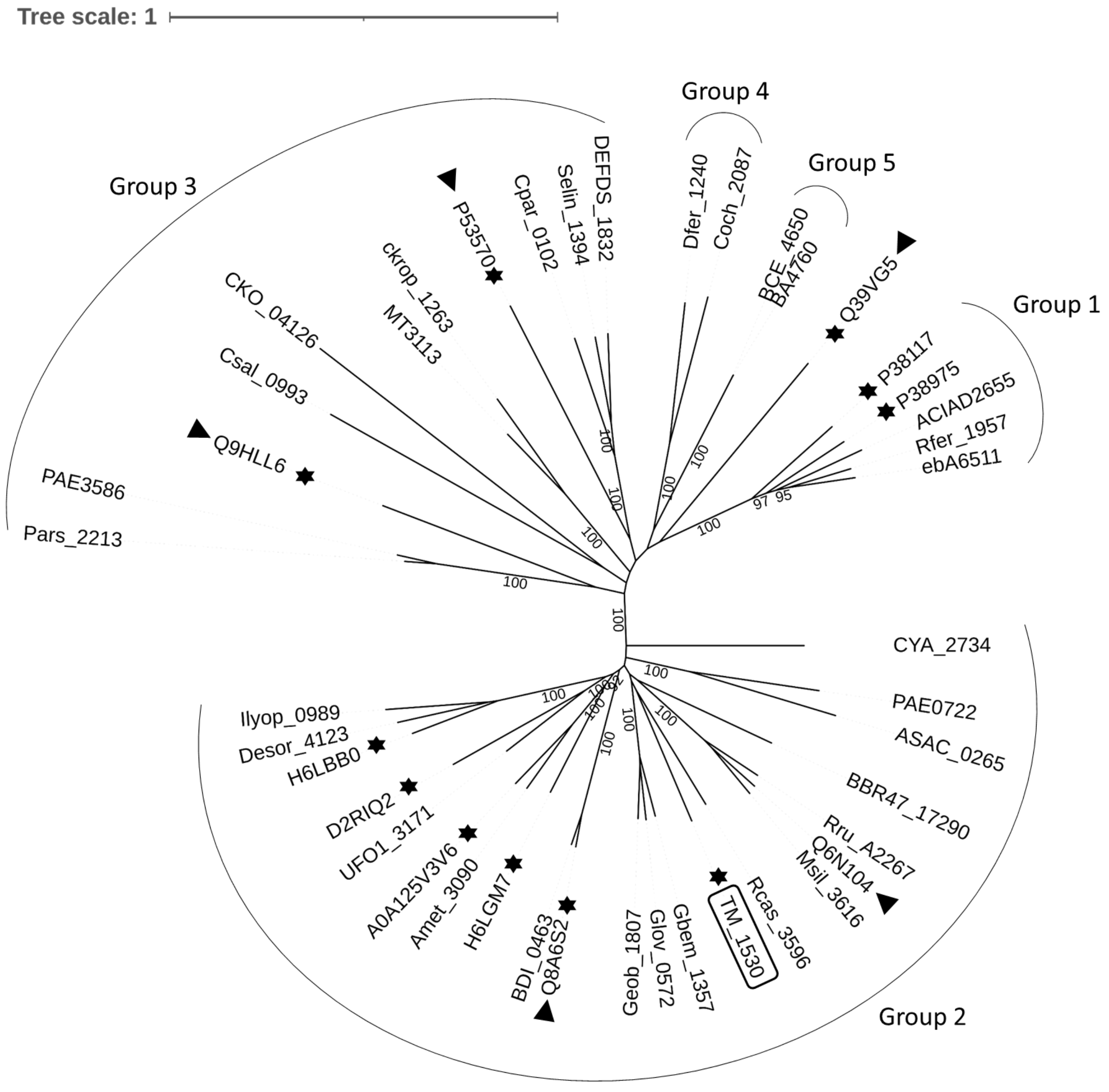

Tree scale: 1
Group 1
Group 2
Group 3
Group 4
Group 5
DEFDS_1832
Selin_1394
Cpar_0102
P53570
ckrop_1263
MT3113
CKO_04126
Csal_0993
Q9HLL6
PAE3586
Pars_2213
Dfer_1240
Coch_2087
BCE_4650
BA4760
Q39VG5
P38117
P38975
ACIAD2655
Rfer_1957
ebA6511
CYA_2734
PAE0722
ASAC_0265
BBR47_17290
Rru_A2267
Q6N104
Msil_3616
Rcas_3596
TM_1530
Gbem_1357
Glov_0572
Geob_1807
Q8A6S2
BDI_0463
H6LGM7
Amet_3090
A0A125V3V6
UFO1_3171
D2RIQ2
H6LBB0
Desor_4123
Ilyop_0989

Figure S5. Multiple alignments of *T. maritima* EtfA and EtfB with their homologs. Homologous sequences listed in **Supplementary Table S2A** and **S2B** were aligned individually using Clustal Omega. All sequences were renamed by their NCBI locus tag. (A) Multi-alignments of Tma EtfA (Tm_1530, highlighted in light grey) and homologs. Conserved amino acid residues Arg, Pro and Val in group 2 sequences, corresponding to Arg38, Pro239 and Val242 in Tma EtfA around the BF-FAD, were marked with red box. (B) Multi-alignments of Tma EtfB (Tm_1531, highlighted in light grey) and homologs. Conserved amino acid residues Arg in group 2 sequences were also marked with red box and this Arg was corresponding to Arg 140 near the BF-FAD. Phylogenetic group of each sequence were indicated on the left side of figure.

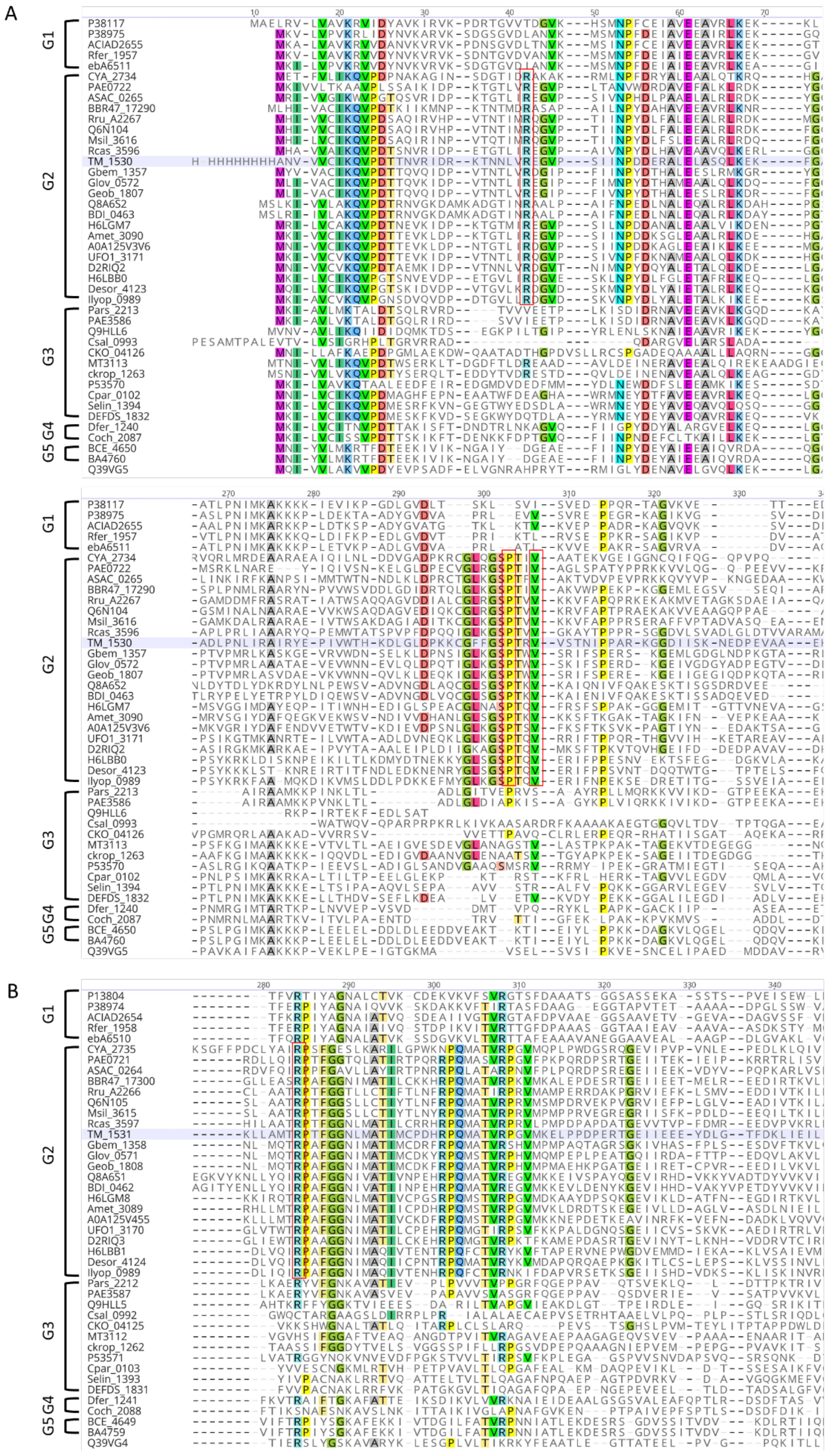
A
G1
G2
G3
G4
G5
P38117
P38975
ACIAD2655
Rfer_1957
ebA6511
CYA_2734
PAE0722
ASAC_0265
BBR47_17290
Rru_A2267
Q6N104
Msil_3616
Rcas_3596
TM_1530
Gbem_1357
Glov_0572
Geob_1807
Q8A652
BDI_0463
H6LGM7
Amet_3090
A0A125V3V6
UFO1_3171
D2RIQ2
H6LBB0
Desor_4123
Ilyop_0989
Pars_2213
PAE3586
Q9HLL6
Csal_0993
CKO_04126
MT3113
ckrop_1263
P53570
Cpar_0102
Selin_1394
DEFDS_1832
Dfer_1240
Coch_2087
BCE_4650
BA4760
Q39VG5
B
P13804
P38974
ACIAD2654
Rfer_1958
ebA6510
CYA_2735
PAE0721
ASAC_0264
BBR47_17300
Rru_A2266
Q6N105
Msil_3615
Rcas_3597
TM_1531
Gbem_1358
Glov_0571
Geob_1808
Q8A651
BDI_0462
H6LGM8
Amet_3089
A0A125V455
UFO1_3170
D2RIQ3
H6LBB1
Desor_4124
Ilyop_0989
Pars_2212
PAE3587
Q9HLL5
Csal_0992
CKO_04125
MT3112
ckrop_1262
P53571
Cpar_0103
Selin_1393
DEFDS_1831
Dfer_1241
Coch_2088
BCE_4649
BA4759
Q39VG4

Figure S6. Non-bifurcating activities and FAD occupancy of EtfAB. The assay contained the wild type and mutant forms of purified EtfAB (20 µg) in 50 mM HEPES, pH 7.5, 100 mM NaCl buffer with 0.5 mM NADH and 0.2 mM iodonitrotetrazolium chloride in anaerobic cuvettes. Reactions were started by injecting NADH. Specific activities are expressed in U/mg where 1 U represents 1 µmole of INT reduced per minute (blue bars). The orange curve indicates the FAD content in each EtfAB complex.

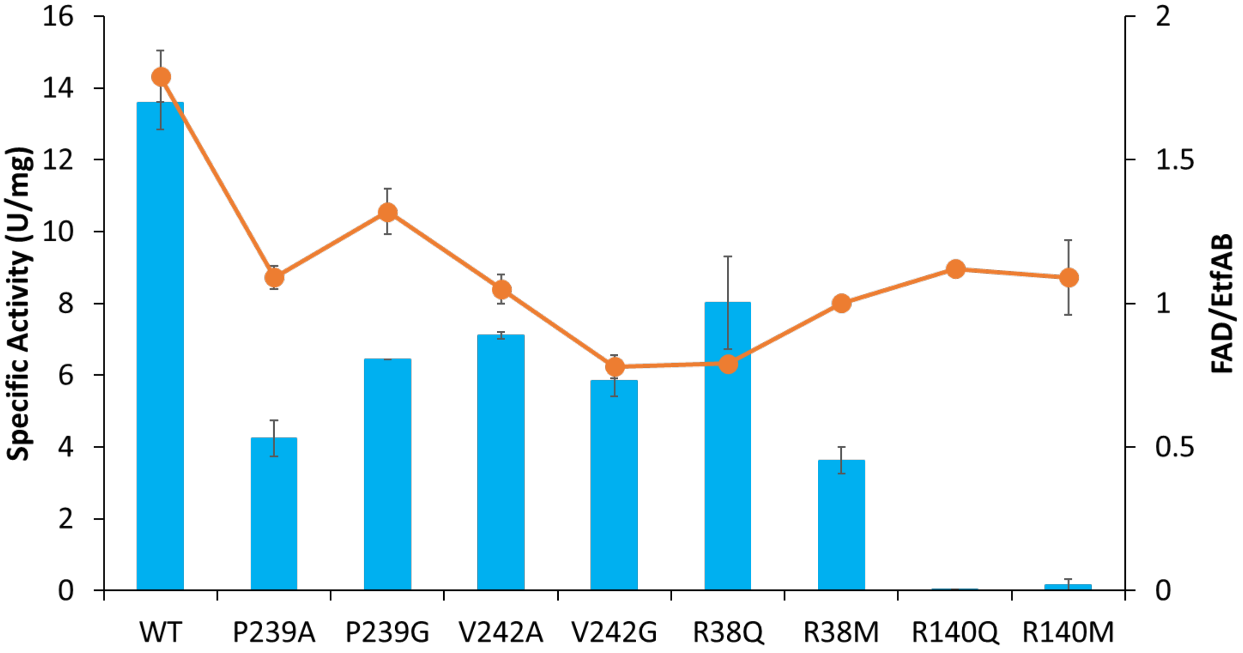

Figure S7. NADH titrations of EtfAB mutants. The mutants are: (A) P239G with 1.32 ± 0.08 FAD, (B) V242G with 0.78 ± 0.04 FAD, (C) R38Q with 0.79 ± 0.02 FAD, (D) R140Q with 1.12 ± 0.02 and (E) R140M with 1.09 ± 0.13. Each mutant (30 μM) was air oxidized and then titrated by NADH in anaerobic cuvettes. Absorbance decreased at 374 nm and 450 nm indicating the reduction of FAD in each mutant.

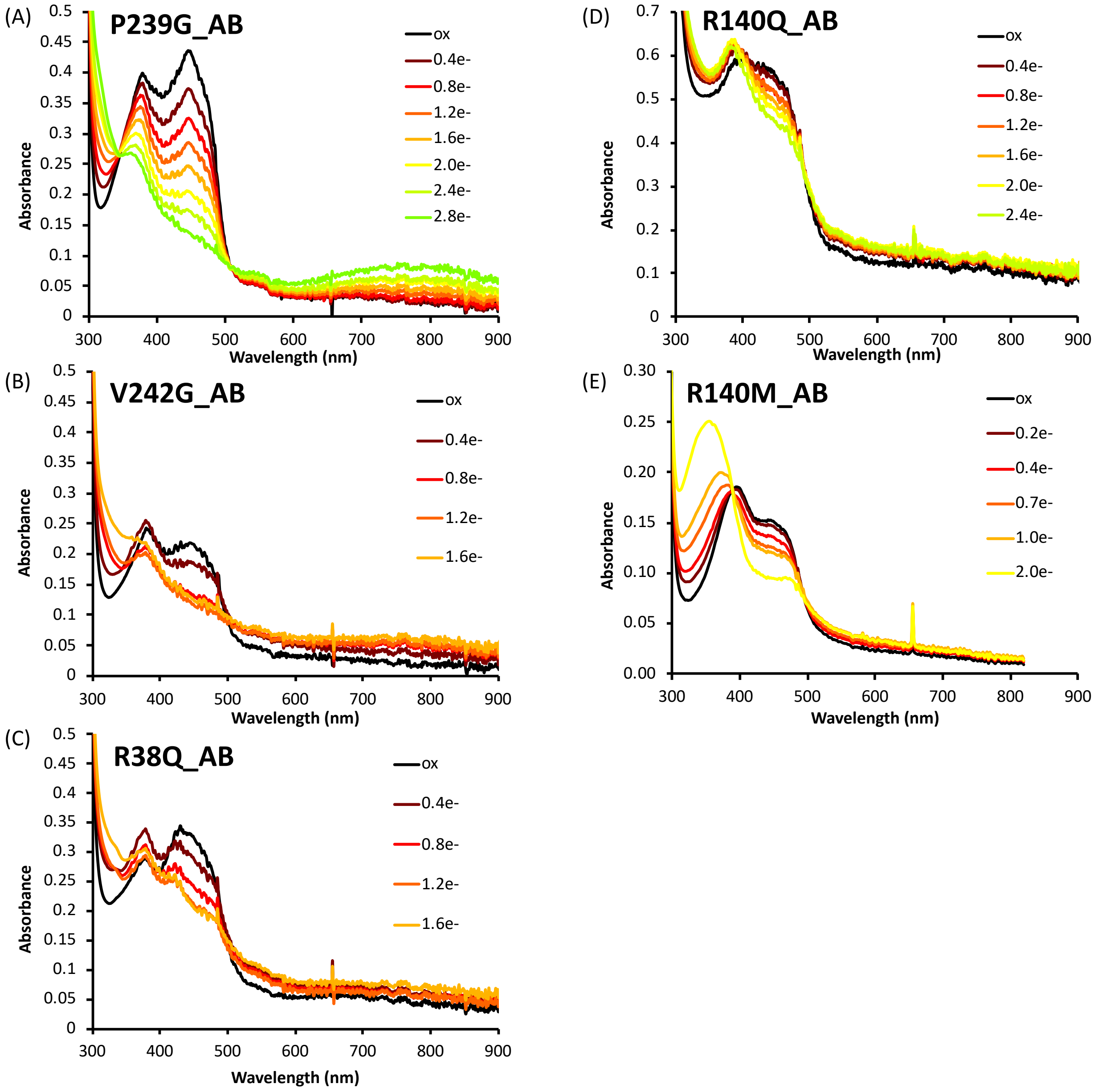

Figure S8. **Deconvolution of the EPR signals seen with 23.1 μM dithionite-reduced R140M EtfABCX.** (A) The experimental spectrum (black) and corresponding simulation (red) seen with dithionite-reduced EtfABCX.The individual components of the simulated spectra attributed to EtfABCX are (B) [4Fe-4S]-I cluster, (C) [4Fe-4S]-II cluster, and (D) anionic semiquinone. The reaction buffer used was 50 mM HEPES, 200 mM NaCl, pH 7.5.

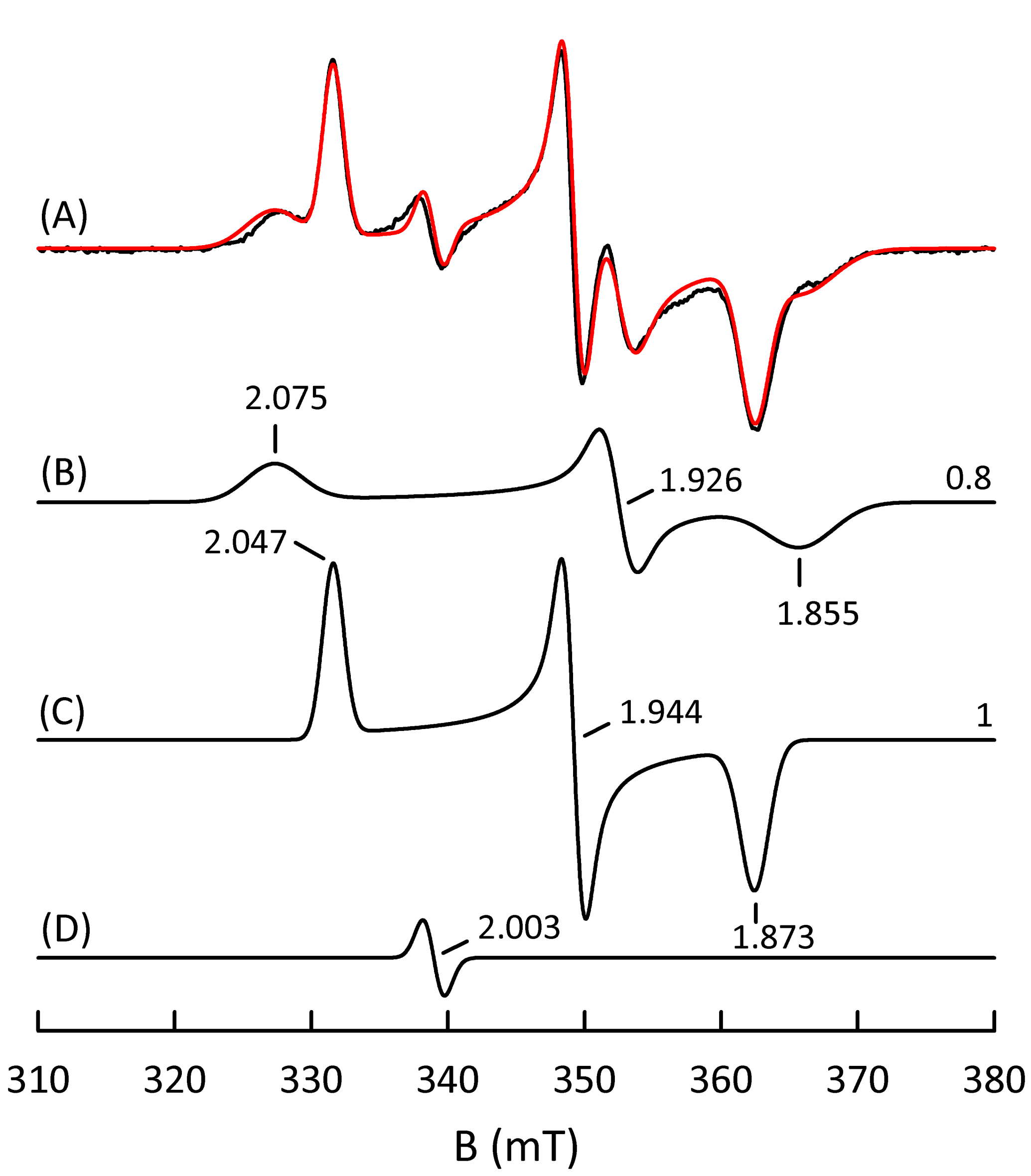

Figure S9. Estimate of the extent of ferredoxin reduction with R140M. (A) The EPR spectrum seen with 23.1 µM R140M EtfABCX reduced by 480 µM NADH. The large isotropic signal in the middle of the spectrum is due to the presence of flavin semiquinone in EtfABCX. (B) the spectrum seen with 23.1 µM EtfABCX, 480 µM NADH in the presence of 120 µM ferredoxin. (C) spectrum of a 21.4 µM reduced ferredoxin standard (red) overlapped with the difference spectrum of (B) minus (A) (black). The small derivative feature in the difference spectrum is due to variable amounts of the semiquinone in the two samples. The amount of ferredoxin reduced was estimated by taking the ratio of the g_1-values from the reduced ferredoxin standard and the difference spectrum. The reaction was performed in 50 mM HEPES, 200 mM NaCl, pH 7.5 at 25°C. The amount of ferredoxin reduced is presented in Table 1. The signals were expanded to clearly visualize the features, cutting off those of the anionic semiquinone.

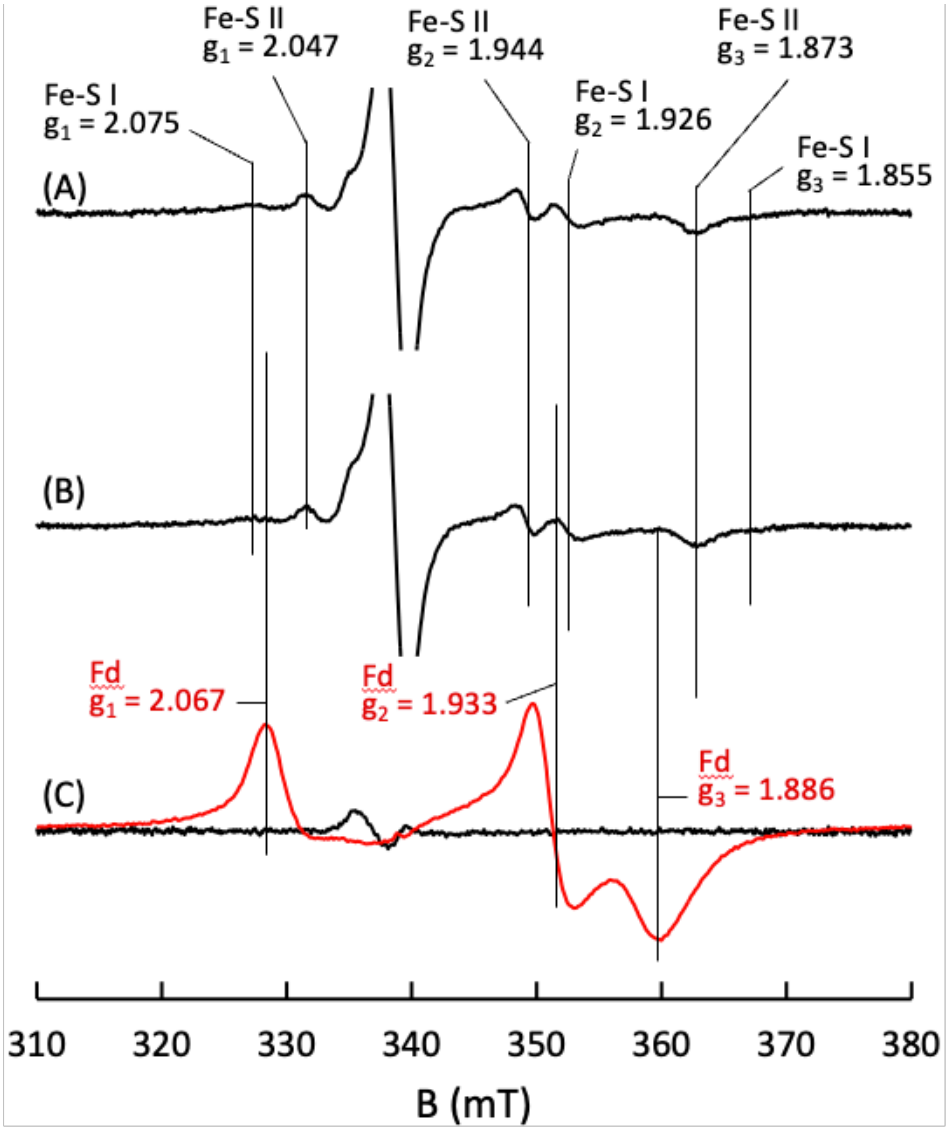
Fe-S I
g1 = 2.075
Fe-S II
g1 = 2.047
Fe-S II
g2 = 1.944
Fe-S I
g2 = 1.926
Fe-S II
g3 = 1.873
Fe-S I
g3 = 1.855
(A)
(B)
(C)
Fd
g1 = 2.067
Fd
g2 = 1.933
Fd
g3 = 1.886
310
320
330
340
350
360
370
380
B (mT)

Figure S10. Deconvolution of the EPR signals seen with dithionite-reduced R38Q EtfABCX. (A) The experimental spectrum (black) and corresponding simulation (red) seen with dithionite-reduced EtfABCX (23.1 μM). The individual components of the simulated spectra attributed to EtfABCX are (B) [4Fe-4S]-I cluster, (C) [4Fe-4S]-II cluster, and (D) anionic semiquinone. The reaction buffer used was 50 mM HEPES, 200 mM NaCl, pH 7.5.

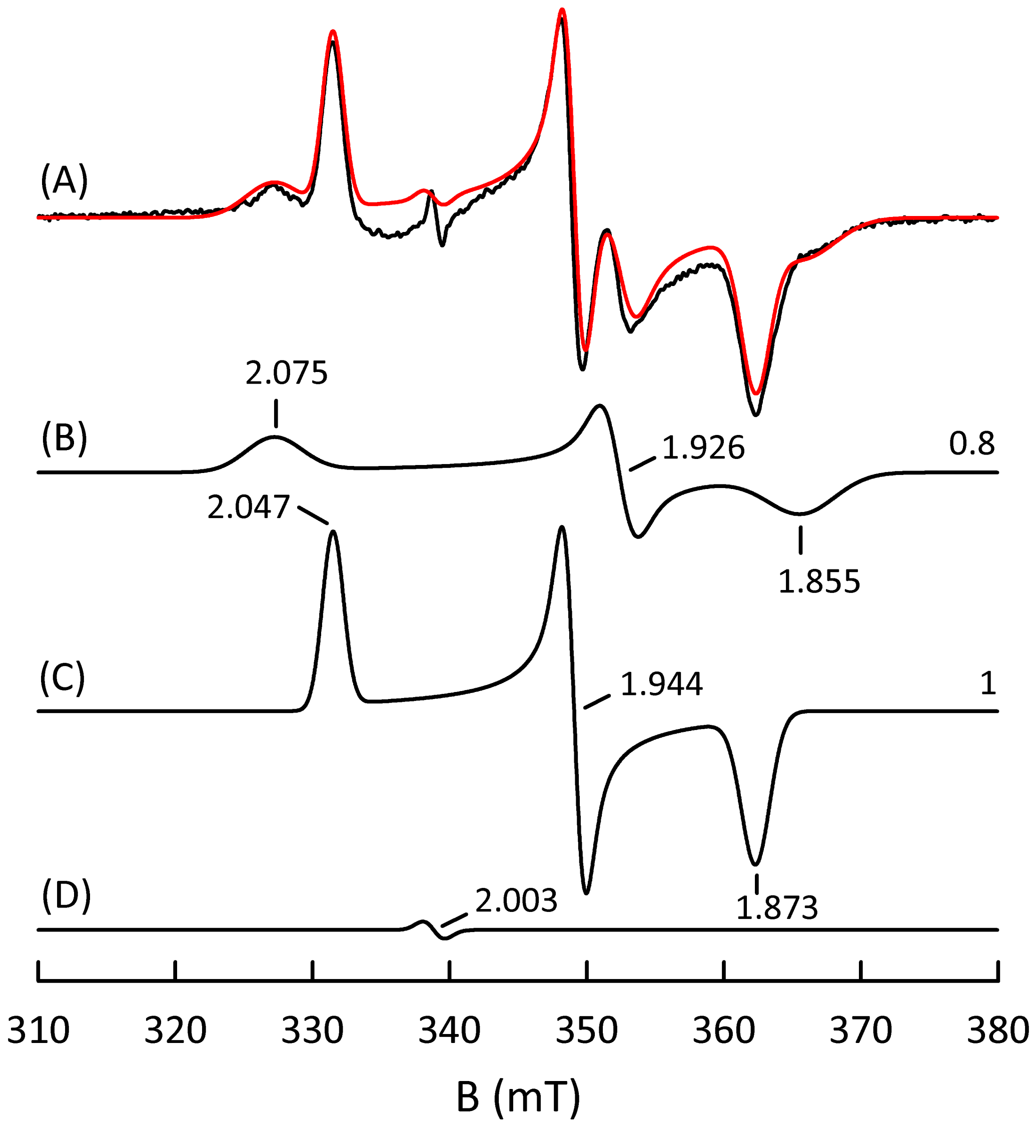

Figure S11. Estimate of the extent of ferredoxin reduction with R38Q. (A) The EPR spectrum seen with R38Q EtfABCX (23.1 μM) reduced by NADH (480 μM). The large isotropic signal in the middle of the spectrum is due to the presence of flavin semiquinone in EtfABCX. (B) The spectrum seen with EtfABCX (23.1 μM), NADH (480 μM) in the presence of ferredoxin (120 μM). (C) spectrum of reduced ferredoxin (21.4 μM, red) overlapped with the difference spectrum of (B) minus (A) (black). The small inverted derivative feature in the difference spectrum is due to variable amounts of the semiquinone in the two samples. The amount of ferredoxin reduced was estimated by taking the ratio of the g_1-values from the reduced ferredoxin standard and the difference spectrum. The reaction was performed in 50 mM HEPES, 200 mM NaCl, pH 7.5 at 25°C. The amount of ferredoxin reduced is presented in Table 1. The signals were expanded to clearly visualize the features, cutting off those of the anionic semiquinone.

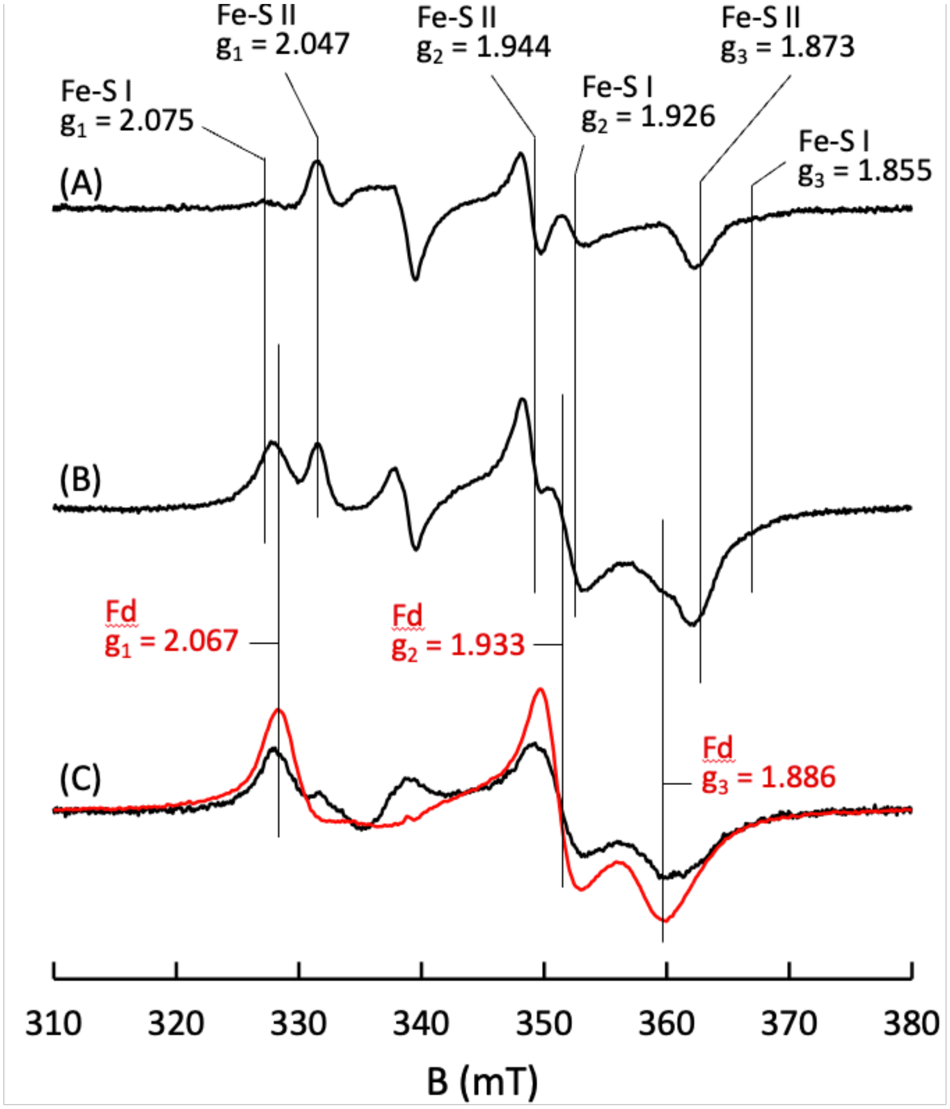

Fe-S II
g_1 = 2.047
Fe-S II
g_2 = 1.944
Fe-S II
g_3 = 1.873
Fe-S I
g_1 = 2.075
Fe-S I
g_2 = 1.926
Fe-S I
g_3 = 1.855
(A)
(B)
Fd
g_1 = 2.067
Fd
g_2 = 1.933
Fd
g_3 = 1.886
(C)
310
320
330
340
350
360
370
380
B (mT)

Figure S12. Deconvolution of the EPR signals seen with 23.1 µM dithionite-reduced P239G EtfABCX. (A) The experimental spectrum (black) and corresponding simulation (red) seen with dithionite-reduced EtfABCX. The individual components of the simulated spectra attributed to EtfABCX are (B) [4Fe-4S]-I cluster, (C) [4Fe-4S]-II cluster, and (D) anionic semiquinone. The reaction buffer used was 50 mM HEPES, 200 mM NaCl, pH 7.5.

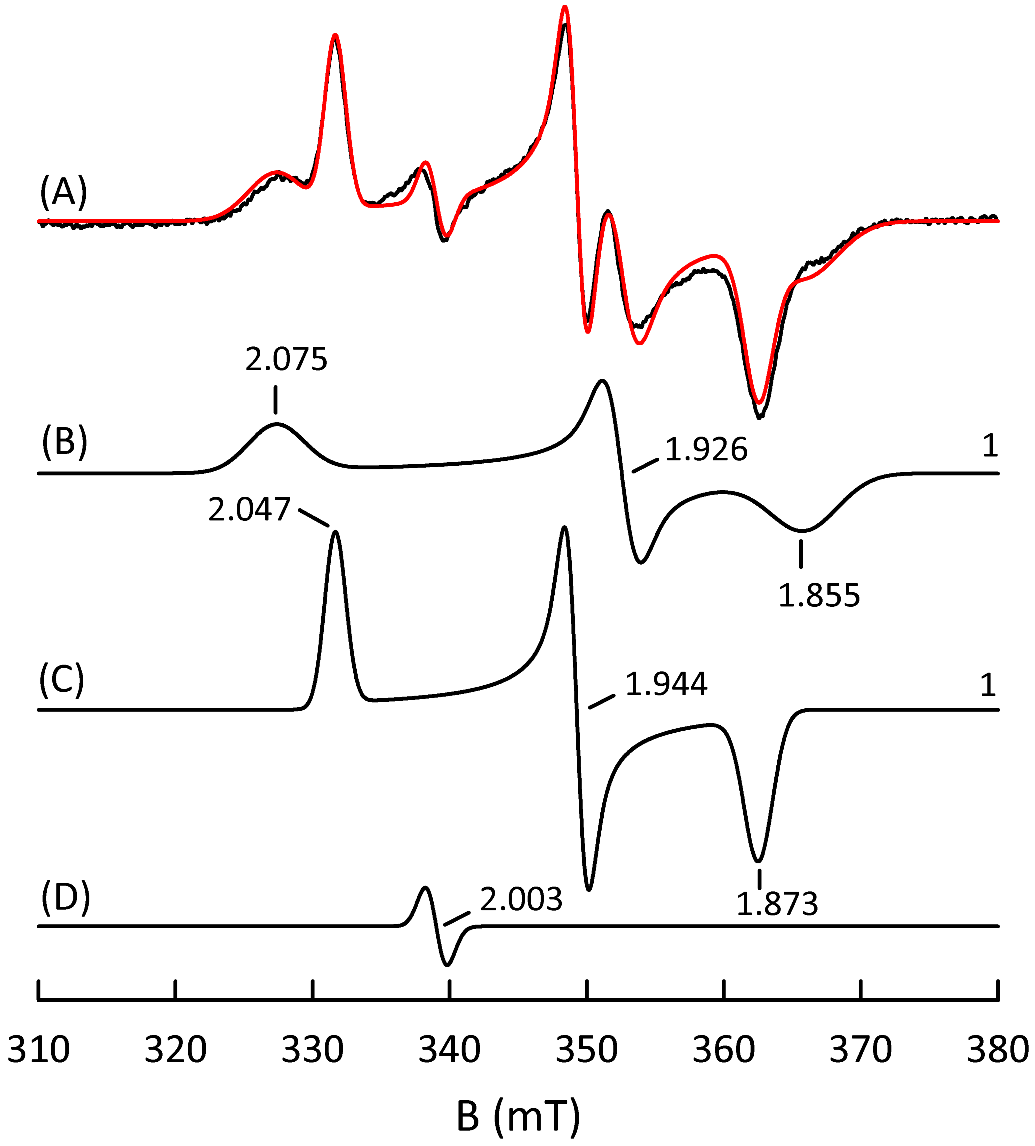

Figure S13. Estimate of the extent of ferredoxin reduction with P239G. (A) The EPR spectrum seen with P239G EtfABCX (23.1 μM) reduced by NADH (480 μM). The large isotropic signal in the middle of the spectrum is due to the presence of flavin semiquinone in EtfABCX. (B) The spectrum seen with P239G EtfABCX (23.1 μM) reduced by NADH (480 μM) in the presence of ferredoxin (120 μM). (C) The spectrum of reduced ferredoxin (21.4 μM, red) overlapped with the difference spectrum of (B) minus (A) (black). The large inverted derivative feature in the difference spectrum is due to variable amounts of the semiquinone in the two samples. The amount of ferredoxin reduced was estimated by taking the ratio of the g_1-values from the reduced ferredoxin standard and the difference spectrum. The reaction was performed in 50 mM HEPES, 200 mM NaCl, pH 7.5 at 25°C. The amount of ferredoxin reduced is presented in Table 1. The signals were expanded to clearly visualize the features, cutting off those of the anionic semiquinone.

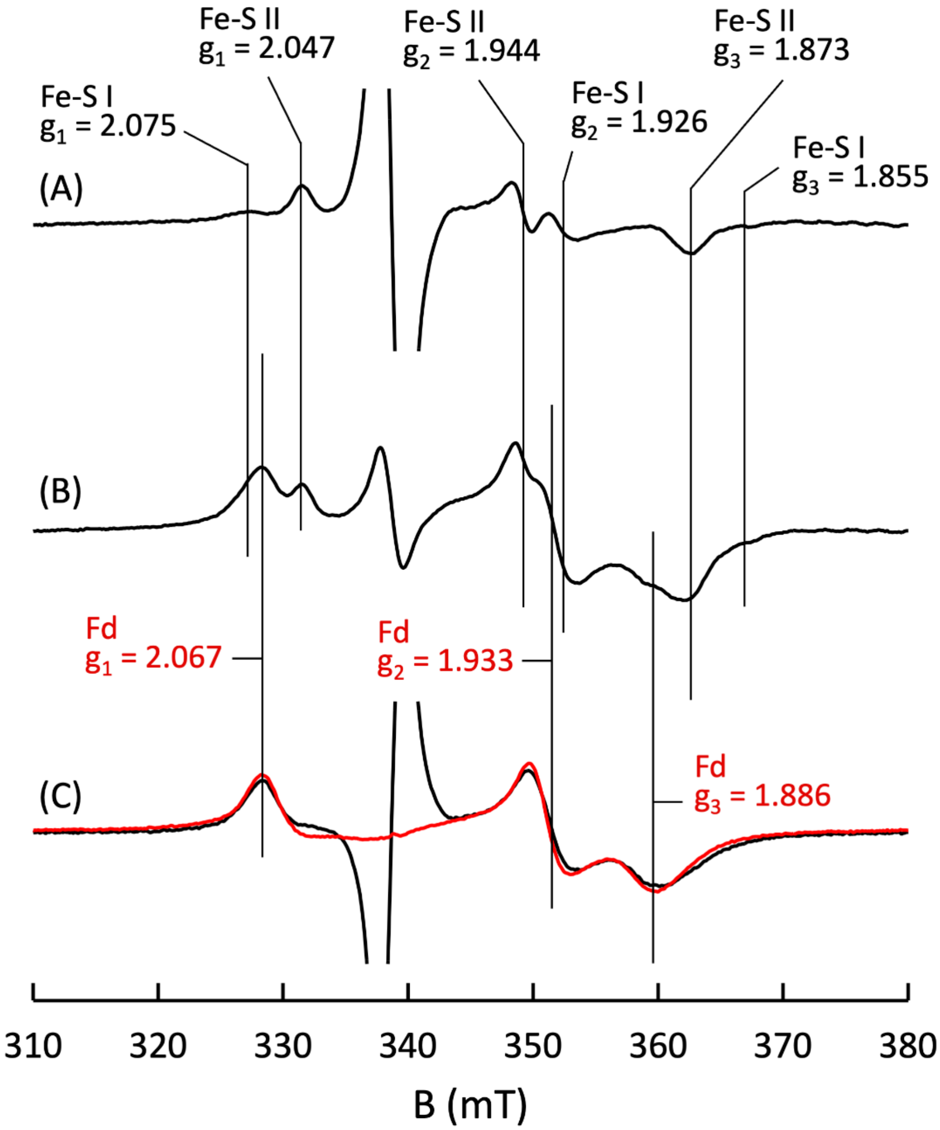

Fe-S II
g_1 = 2.047
Fe-S II
g_2 = 1.944
Fe-S II
g_3 = 1.873
Fe-S I
g_1 = 2.075
Fe-S I
g_2 = 1.926
Fe-S I
g_3 = 1.855
(A)
(B)
Fd
g_1 = 2.067
Fd
g_2 = 1.933
Fd
g_3 = 1.886
(C)
310
320
330
340
350
360
370
380
B (mT)

Figure S14. Deconvolution of the EPR signals seen with dithionite-reduced V242G EtfABCX. (A) The experimental spectrum (black) and corresponding simulation (red) seen with dithionite-reduced V242G EtfABCX (23.1 μM). The individual components of the simulated spectra attributed to EtfABCX are (B) [4Fe-4S]-I cluster, (C) [4Fe-4S]-II cluster, and (D) anionic semiquinone. The reaction buffer used was 50 mM HEPES, 200 mM NaCl, pH 7.5.

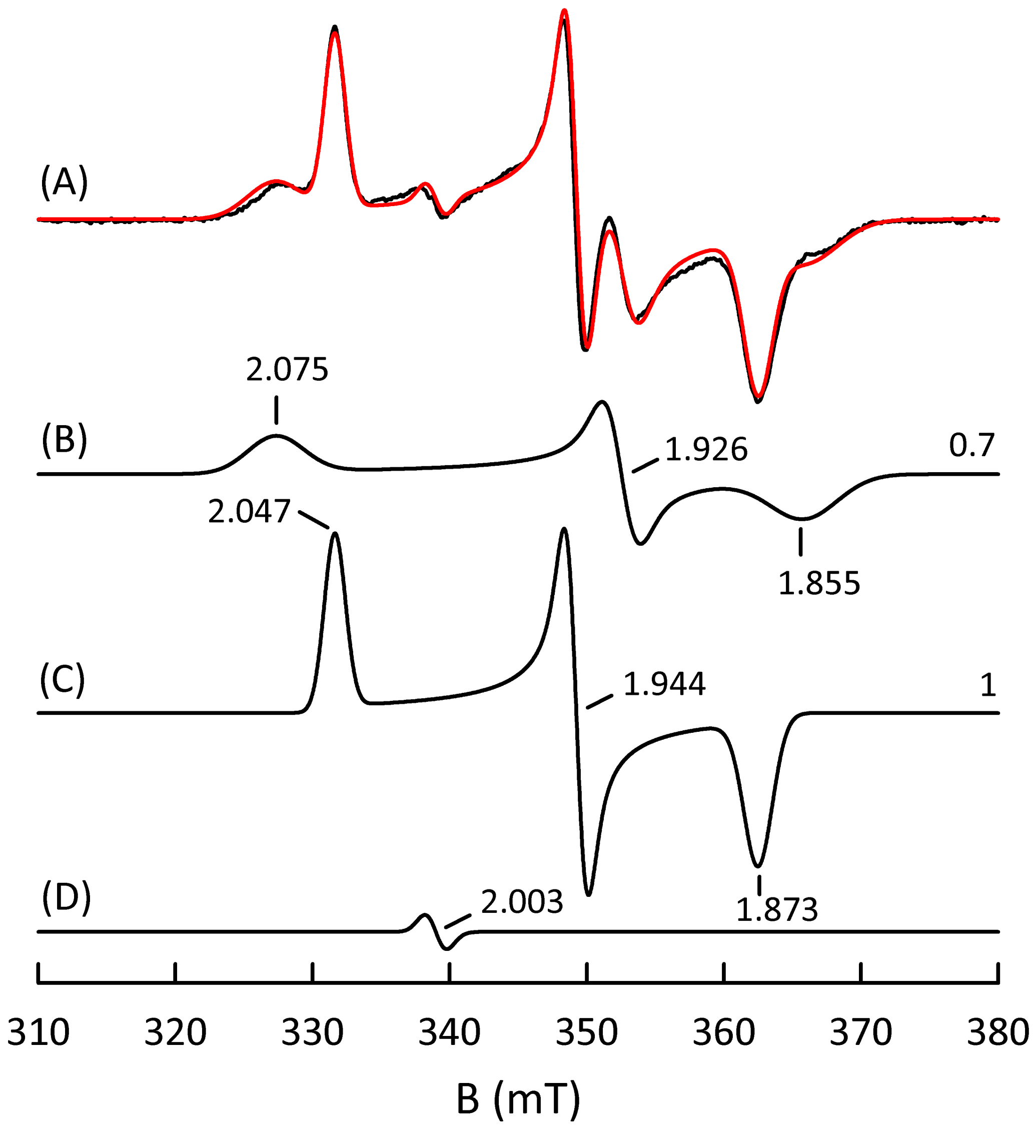

Figure S15. Estimate of the extent of ferredoxin reduction with V242G. (A) The EPR spectrum seen with V242G EtfABCX (23.1 μM) reduced by NADH (480 μM). The large isotropic signal in the middle of the spectrum is due to the presence of flavin semiquinone in EtfABCX. (B) The spectrum seen with V242G EtfABCX (23.1 μM) reduced by NADH (480 μM) in the presence of ferredoxin (120 μM). (C) The spectrum of reduced ferredoxin standard (21.4 μM, red) overlapped with the difference spectrum of (B) minus (A) (black). The large inverted derivative feature in the difference spectrum is due to variable amounts of the semiquinone in the two samples. The amount of ferredoxin reduced was estimated by taking the ratio of the g_1-values from the reduced ferredoxin standard and the difference spectrum. The reaction was performed in 50 mM HEPES, 200 mM NaCl, pH 7.5 at 25°C. The amount of ferredoxin reduced is presented in Table 1. The signals were expanded to clearly visualize the features, cutting off those of the anionic semiquinone.

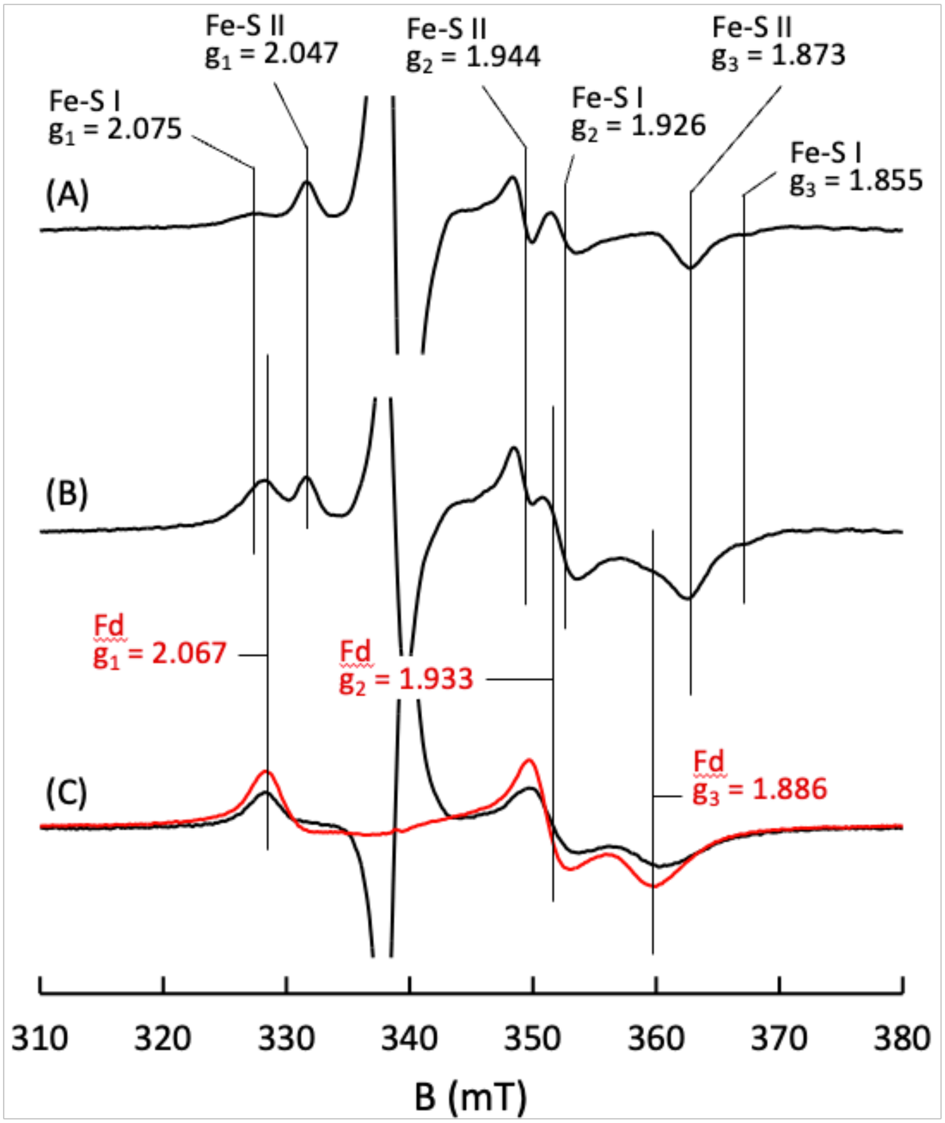

Fe-S II
g1 = 2.047
Fe-S II
g2 = 1.944
Fe-S II
g3 = 1.873
Fe-S I
g1 = 2.075
Fe-S I
g2 = 1.926
Fe-S I
g3 = 1.855
(A)
(B)
Fd
g1 = 2.067
Fd
g2 = 1.933
Fd
g3 = 1.886
(C)
310
320
330
340
350
360
370
380
B (mT)

Figure S16. UV-visible spectrum of as-purified (A) WT EtfAB and (B) WT EtfABCX. Blue arrows indicate the absorbance at 374 nm, orange arrows indicate the absorbance at 450 nm.

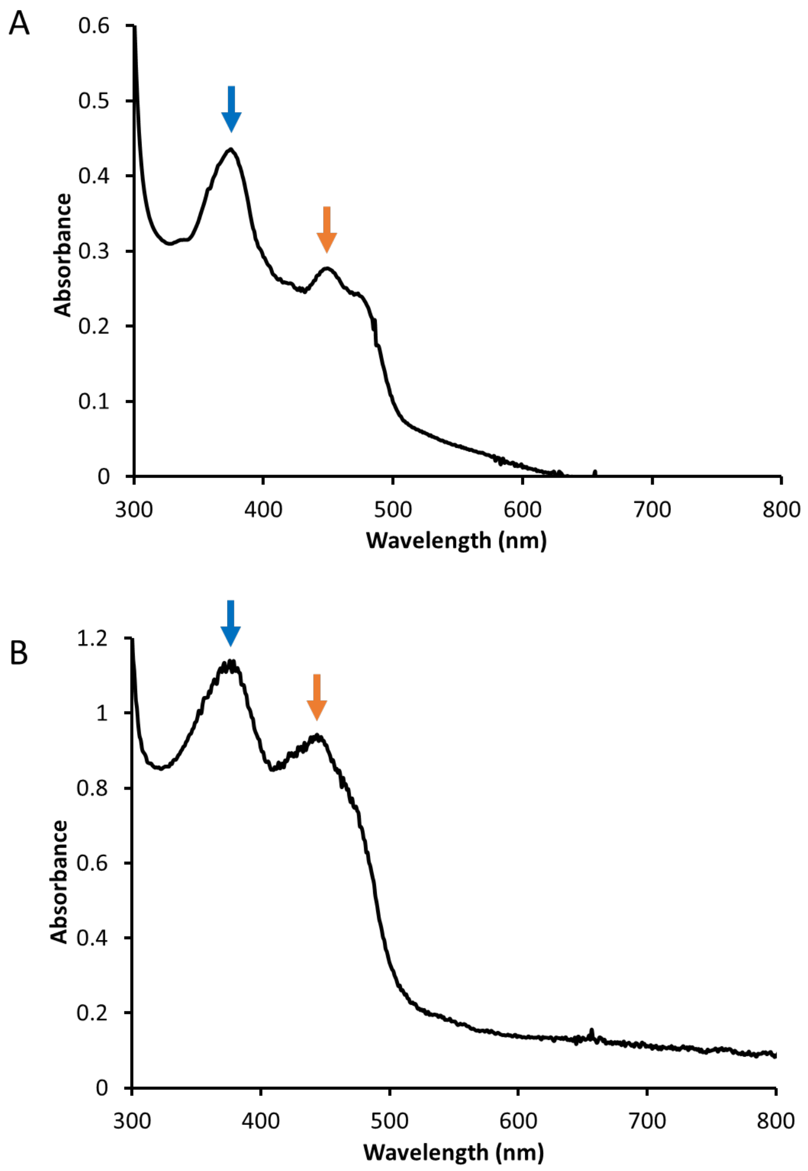

Figure S17. NAD$^+$ titration of Ti-reduced EtfAB. WT EtfAB (50 µM) was fully reduced by Ti (III) in an anaerobic cuvette and then titrated with NAD$^+$. Black arrow indicates the formation of the charge transfer band (550-900 nm).

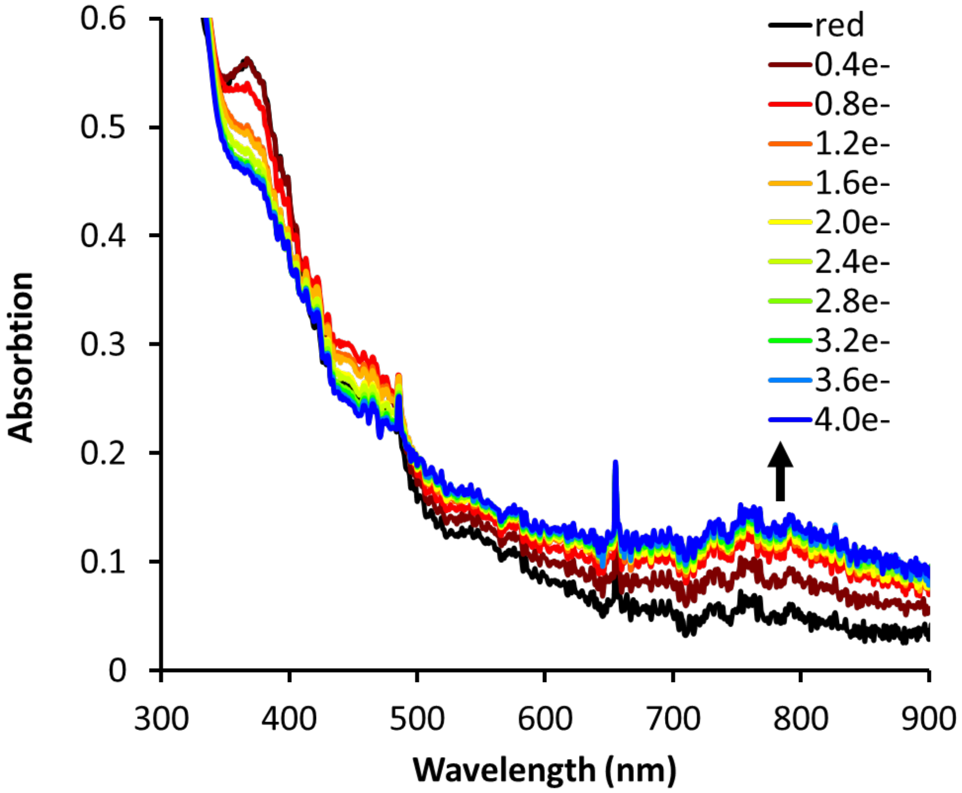

Figure S18. NADH and Ti titration of EtfABCX R140M. (A) Air-oxidized R140M EtfABCX (15 μM) containing 2.59 ± 0.09 of FAD/mole was titrated by NADH to 8 e- equivalents and then further titrated by Ti (III) to 11 e- equivalents. The inset to the figure indicates absorbance changes at 374 nm and 450 nm of R140M EtfABCX titration. (B) Difference spectrum of EtfABCX R140M. (C) Air-oxidized R140M EtfABCX (15 μM) was titrated by Ti (III) to 11 e- equivalents.

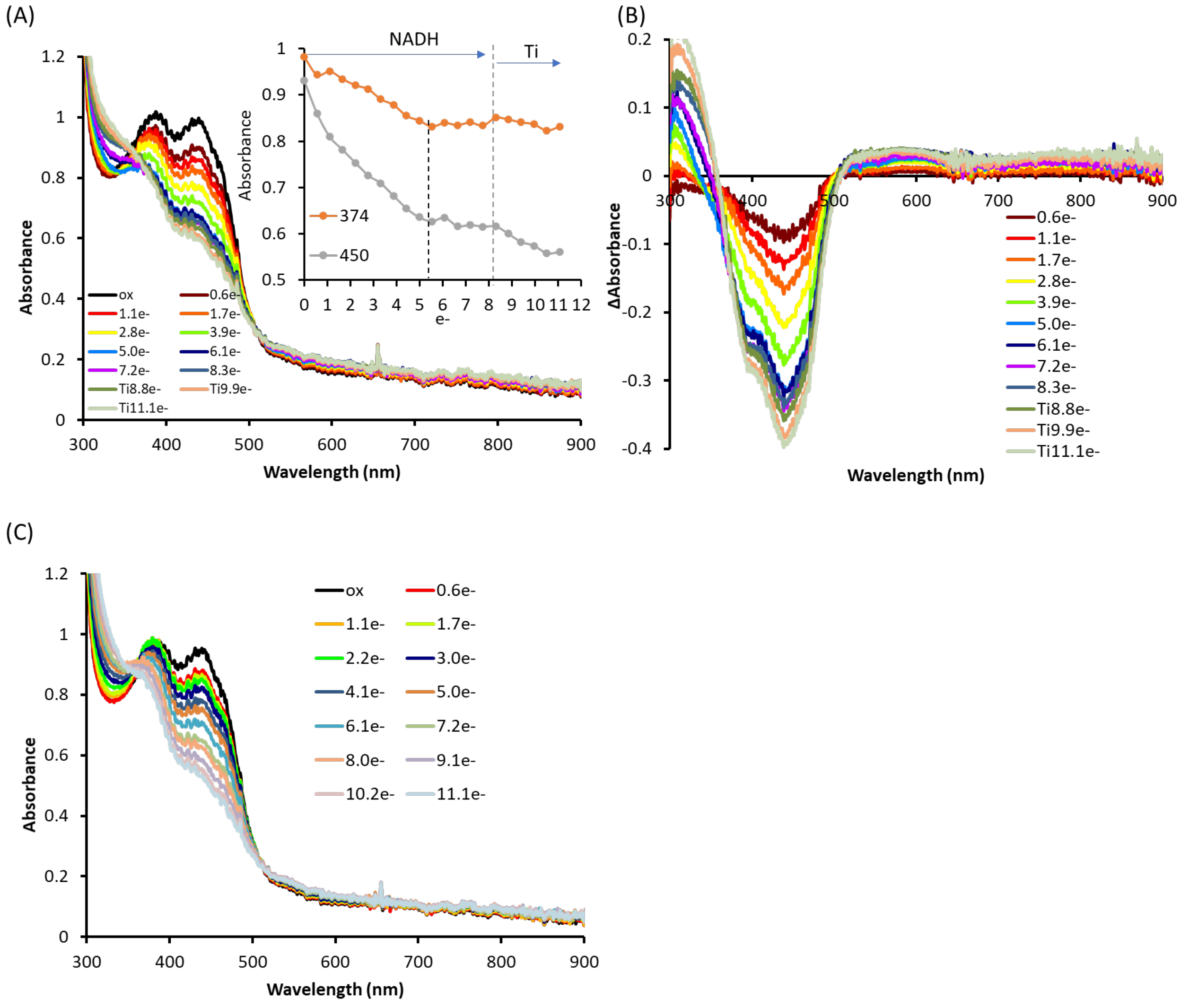

Figure S19. Proposed impact of the R140 mutation on the mechanism of electron bifurcation. The mechanism is based on Figure. 6 of ref. 1. Each step in the mechanism indicates the number of bound electrons the complex contains and whether they are of low, median (mid) or high potential. The complete catalytic cycle has 10 steps with 10 intermediate catalytic states but states 6-10 , in which two additional electrons are added by the oxidation of a second NADH, are not shown here for simplicity. Based on the spectroscopic data presented here, the mechanisms of the wild-type (WT) and the R140 mutants diverge between step 2 and step 3, after the oxidized ferredoxin (Fd) binds. Namely, in step 3A the BF-FAD is no longer bifurcating, the low-potential electron is not generated and ferredoxin is not reduced, and the reaction cannot proceed.

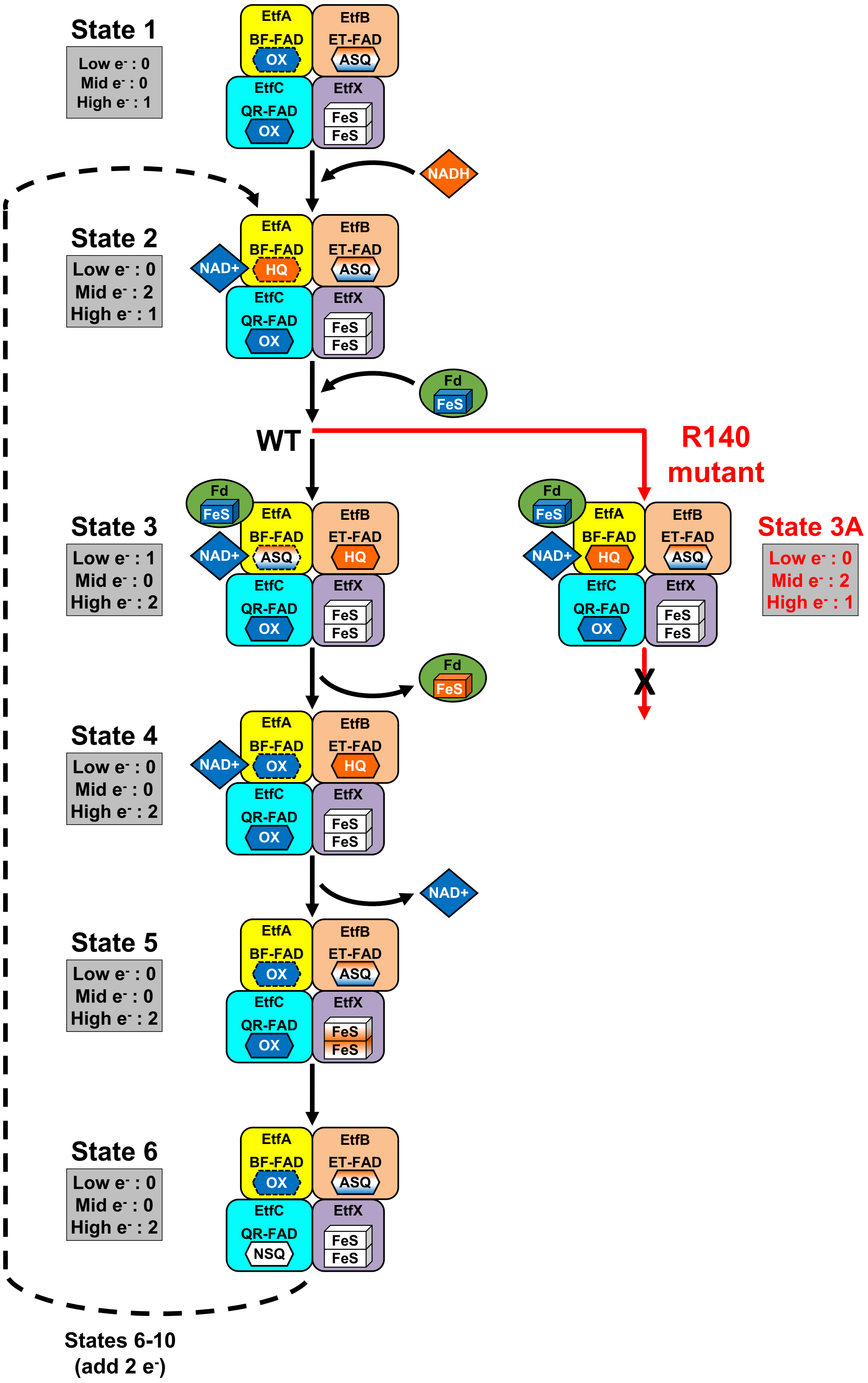
State 1
Low e⁻ : 0
Mid e⁻ : 0
High e⁻ : 1
EtfA
BF-FAD
OX
EtfB
ET-FAD
ASQ
EtfC
QR-FAD
OX
EtfX
FeS
FeS
NADH
State 2
Low e⁻ : 0
Mid e⁻ : 2
High e⁻ : 1
NAD+
HQ
Fd
FeS
WT
R140
mutant
State 3
Low e⁻ : 1
Mid e⁻ : 0
High e⁻ : 2
State 3A
Low e⁻ : 0
Mid e⁻ : 2
High e⁻ : 1
X
State 4
Low e⁻ : 0
Mid e⁻ : 0
High e⁻ : 2
State 5
Low e⁻ : 0
Mid e⁻ : 0
High e⁻ : 2
State 6
Low e⁻ : 0
Mid e⁻ : 0
High e⁻ : 2
NSQ
States 6-10
(add 2 e⁻)

Table S1. Primers used in this study

Note	Sequence (5' to 3')	Target Gene
pET21a-F	TGAGATCCGGCTGCTAACAAAGCC	pET21a vector backbone
pET21a-R	CATATGTATATCTCCTTCTTAAAGTTAAAC	pET21a vector backbone
Tma-EtfABCX-F	GTTTAACTTTAAGAAGGAGATATACATATGGCACATCACCACCATCACCACC	Tma-*etfABCX*
Tma-EtfABCX-R	GGCTTTGTTAGCAGCCGGATCTCATCACCCGAATTTGTAGAGCAC	Tma-*etfABCX*
Tma-Fd-F	GTTTAACTTTAAGAAGGAGATATACATATGAAGGTAAGAGTTGACGCAGATGC	Tma-*Fd*
Tma-Fd-R	GGCTTTGTTAGCAGCCGGATCTCATCACTCTTCTACGCTGATAGCTC	Tma-*Fd*
AP239A-FL	CAAACATACCCCCGGCGAGAAAAGGTG	*etfA*
AP239A-RS	CCAAAGAATCCACATTTTTTTGGATCG	*etfA*
AP239A-RL	TGCTCACAACCCTTGTCGCTGATCCAAAGAATCCACATTTTTTTGG	*etfA*
AP239A-FS	CAAACATACCCCCGGCGAGAAAAGGTG	*etfA*
AP239G-FL	ATCAGGCACAAGGGTTGTGAGCACAAACATACCCCCGGCGAGAAAAG	*etfA*
AP239G-RL	TGCTCACAACCCTTGTGCCTGATCCAAAGAATCCACATTTTTTTGG	*etfA*
AV242A-FL	ATCACCTACAAGGGCGGTGAGCACAAACATACCCCCGGCGAGAAAAG	*etfA*
AV242A-RL	TGCTCACCGCCCTTGTAGGTGATCCAAAGAATCCACATTTTTTTGG	*etfA*
AV242G-FL	ATCACCTACAAGGGGCGTGAGCACAAACATACCCCCGGCGAGAAAAG	*etfA*
AV242G-RL	TGCTCACGCCCCTTGTAGGTGATCCAAAGAATCCACATTTTTTTGG	*etfA*
AR38Q-FL	CTTGTGCAGGAAGGTGTCCCGTCTATCATAAATCC	*etfA*
AR38Q-RS	GTTGTTCGTTTTTCTGTCGATTC	*etfA*
AR38Q-RL	CGGGACACCTTCCTGCACAAGGTTGTTCGTTTTTCTGTC	*etfA*
AR38Q-FS	TCTATCATAAATCCCGATGATG	*etfA*
AR38M-FL	CTTGTGATGGAAGGTGTCCCGTCTATCATAAATCC	*etfA*
AR38M-RL	CGGGACACCTTCCATCACAAGGTTGTTCGTTTTTCTGTC	*etfA*
BR140Q-FL	ATGACGCAGCCCACATTTGGTGGAAACCTCATGGCAACC	*etfB*
BR140Q-RS	GGCAAGAAGCTTTTTGTCCGG	*etfB*
BR140Q-RL	ACCAAATGTGGGCTGCGTCATGGCAAGAAGCTTTTTGTC	*etfB*
BR140Q-FS	GGAAACCTCATGGCAACCATC	*etfB*
BR140M-FL	ATGACGATGCCCACATTTGGTGGAAACCTCATGGCAACC	*etfB*
BR140M-RL	ACCAAATGTGGGCATCGTCATGGCAAGAAGCTTTTTGTC	*etfB*
TmaXC61A-FL	GGGGCGCTCGAGTGTGGCACCTGTAGGATTG	*etfX*
TmaXC61A-RL	GCCACACTCGAGCGCCCCCTCGAATTTCACC	*etfX*
TmaXC61AC64A-FL	GGGGCGCTCGAGGCGGGCACCTGTAGGATTG	*etfX*
TmaXC61A64A-RL	GCCCGCCTCGAGCGCCCCCTCGAATTTCACC	*etfX*
TmaXC61AC64A-RS	CTCGAATTTCACCTCCATTCCACTCTCCGTC	*etfX*
TmaXC61AC64A-FS	ACCTGTAGGATTGTTTGTCCCTTTGGAAAC	*etfX*
TmaXC29A-FL	ATCGCGGCGGAAAAAATGTTCCGATCGACCATGTGTGAGC	*etfX*
TmaXC29AC33A-RS	GCTTTCATCTTTTATTTTGAGATGCGG	*etfX*
TmaXC29A-RL	GCTCACACATGGTCGATCGGAACATTTTTTCCGCCGCGATGCTTTC	*etfX*
TmaXC29AC33A-FS	TGTTGTCCTGCGGATGTGTACGAG	*etfX*
TmaXC29AC33A-FL	ATCGCGGCGGAAAAAAGCGTCCGATCGACCATGTGTGAGC	*etfX*
TmaXC29AC33A-RL	GCTCACACATGGTCGATCGGACGCTTTTTTCCGCCGCGATGCTTTC	*etfX*

Supplementary Table S2. Description, identity analysis, locus tag, protein name, organism, and sequence for Tma EtfA (A) and EtfB (B) homologs used in this study. Supplied as an Excel file.

Supplementary Table S3. FAD content of EtfAB and EtfABCX mutants.

EtfAB	FAD	% Occupancy	EtfABCX	FAD	% Occupancy
**WT**	1.79 ± 0.09	100.0	**WT**	3.26 ± 0.14	100.0
**R38Q**	0.79 ± 0.02	44.1 ± 1.1	**R38Q**	3.09 ± 0.13	94.6 ± 4.1
**R38M**	1.00 ± 0.01	55.9 ± 0.6	**R38M**	2.42 ± 0.10	74.3 ± 3.1
**P239A**	1.09 ± 0.04	60.9 ± 2.2	**P239A**	2.69 ± 0.01	82.5 ± 0.4
**P239G**	1.32 ± 0.08	73.7 ± 4.5	**P239G**	3.51 ± 0.01	107.5 ± 0.4
**V242A**	1.05 ± 0.05	58.7 ± 2.8	**V242A**	2.49 ± 0.28	76.4 ± 8.7
**V242G**	0.78 ± 0.04	43.6 ± 2.2	**V242G**	2.75 ± 0.11	84.3 ± 3.2
**R140Q**	1.12 ± 0.02	62.6 ± 1.1	**R140Q**	3.44 ± 0.32	105.5 ± 9.8
**R140M**	1.09 ± 0.13	60.9 ± 7.3	**R140M**	2.59 ± 0.09	79.4 ± 2.9

Supplementary Table S4. Fe content of EtfABCX mutants.

EtfABCX	Fe
**WT**	7.1 ± 0.5
**P239G**	7.4 ± 0.1
**V242G**	7.1 ± 0.4
**R38Q**	7.7 ± 0.1
**R140Q**	7.1 ± 0.7
**R140M**	7.8 ± 0.9
**XC29A**	3.1 ± 0.3

References

[1] Feng, X., Schut, G. J., Lipscomb, G. L., Li, H., and Adams, M. W. (2021) Cryoelectron microscopy structure and mechanism of the membrane-associated electron-bifurcating flavoprotein Fix/EtfABCX, *Proc. Natl. Acad. Sci. USA 118*, e2016978118 (doi: 10.1073/pnas. 2016978118)
